# Effect of erythromycin on mortality and the host response in critically ill patients with sepsis: a target trial emulation

**DOI:** 10.1186/s13054-022-04016-x

**Published:** 2022-05-24

**Authors:** Tom D. Y. Reijnders, Hessel Peters-Sengers, Lonneke A. van Vught, Fabrice Uhel, Marc J. M. Bonten, Olaf L. Cremer, Marcus J. Schultz, Martijn M. Stuiver, Tom van der Poll, the MARS consortium, Friso M. de Beer, Lieuwe D. J. Bos, Gerie J. Glas, Roosmarijn T. M. van Hooijdonk, Janneke Horn, Laura R. A. Schouten, Marleen Straat, Luuk Wieske, Esther Witteveen, Tom D. Y. Reijnders, Alex R. Schuurman, Tjitske S. R. van Engelen, Liza Pereverzeva, Arie J. Hoogendijk, Mischa A. Huson, Maryse A. Wiewel, Peter M. C. Klein Klouwenberg, David S. Y. Ong, Jos F. Frencken, Maria E. Koster-Brouwer, Kirsten van de Groep, Diana M. Verboom

**Affiliations:** 1 Center for Experimental and Molecular Medicine, Amsterdam University Medical Centers, Location Academic Medical Center, Meibergdreef 9, 1105 AZ Amsterdam, The Netherlands; 2 AP-HP, Hôpital Louis Mourier, DMU ESPRIT, Médecine Intensive-Réanimation, 92700 Colombes, France; 3 Université de Paris, UFR de Médecine, 75018 Paris, France; 4 INSERM U1151, CNRS UMR 8253, Institut Necker-Enfants Malades, 75015 Paris, France; 5 Department of Medical Microbiology, University Medical Center Utrecht, Utrecht, The Netherlands; 6 Julius Center for Health Sciences and Primary Care, University Medical Center Utrecht, Utrecht, The Netherlands; 7 Department of Intensive Care Medicine, University Medical Center Utrecht, Utrecht, The Netherlands; 8 Department of Intensive Care Medicine, and Laboratory of Experimental Intensive Care and Anesthesiology (LEICA), Amsterdam University Medical Centers, Location Academic Medical Center, Amsterdam, The Netherlands; 9 Mahidol-Oxford Tropical Medicine Research Unit (MORU), Mahidol University, Bangkok, Thailand; 10 Nuffield Department of Medicine, University of Oxford, Oxford, UK; 11 Department of Epidemiology and Data Science, Amsterdam Public Health, Amsterdam University Medical Centers, Location Academic Medical Center, Amsterdam, The Netherlands; 12 Division of Infectious Diseases, Amsterdam University Medical Centers, Location Academic Medical Center, University of Amsterdam, Amsterdam, The Netherlands

**Keywords:** Sepsis, Critically ill, Macrolides, Erythromycin, Immunomodulation, Propensity score, Target trial, Mortality

## Abstract

**Background:**

Immunomodulatory therapies that improve the outcome of sepsis are not available. We sought to determine whether treatment of critically ill patients with sepsis with low-dose erythromycin—a macrolide antibiotic with broad immunomodulatory effects—decreased mortality and ameliorated underlying disease pathophysiology.

**Methods:**

We conducted a target trial emulation, comparing patients with sepsis admitted to two intensive care units (ICU) in the Netherlands for at least 72 h, who were either exposed or not exposed during this period to treatment with low-dose erythromycin (up to 600 mg per day, administered as a prokinetic agent) but no other macrolides. We used two common propensity score methods (matching and inverse probability of treatment weighting) to deal with confounding by indication and subsequently used Cox regression models to estimate the treatment effect on the primary outcome of mortality rate up to day 90. Secondary clinical outcomes included change in SOFA, duration of mechanical ventilation and the incidence of ICU-acquired infections. We used linear mixed models to assess differences in 15 host response biomarkers reflective of key pathophysiological processes from admission to day 4.

**Results:**

In total, 235 patients started low-dose erythromycin treatment, 470 patients served as controls. Treatment started at a median of 38 [IQR 25–52] hours after ICU admission for a median of 5 [IQR 3–8] total doses in the first course. Matching and weighting resulted in populations well balanced for proposed confounders. We found no differences between patients treated with low-dose erythromycin and control subjects in mortality rate up to day 90: matching HR 0.89 (95% CI 0.64–1.24), weighting HR 0.95 (95% CI 0.66–1.36). There were no differences in secondary clinical outcomes. The change in host response biomarker levels from admission to day 4 was similar between erythromycin-treated and control subjects.

**Conclusion:**

In this target trial emulation in critically ill patients with sepsis, we could not demonstrate an effect of treatment with low-dose erythromycin on mortality, secondary clinical outcomes or host response biomarkers.

**Supplementary Information:**

The online version contains supplementary material available at 10.1186/s13054-022-04016-x.

## Introduction

Sepsis—a dysregulated host response to infection culminating in life-threatening organ failure [1]—is a leading global cause of intensive care unit (ICU) admissions and overall in-hospital mortality [2, 3]. Key cellular and physiological disturbances include simultaneous hyperinflammation and immune suppression, endothelial cell dysfunction and coagulopathy [4]. A targeted treatment for sepsis is currently not available, despite the numerous randomized clinical trials (RCTs) that have been conducted with agents that either suppress or stimulate different processes involved in its pathophysiology [5]. Potential therapeutics that inhibit a single pathway or receptor implicated in the septic host response are unlikely to suffice for all patients, because other pathways, receptors and regulatory mechanisms disrupted at the same time may drive pathology and outcome [6].

Macrolides, inhibitors of ribosomal protein synthesis, are a class of antibiotics used to control a broad spectrum of bacterial infections. Some macrolides, like erythromycin, also function as motilin receptor agonists and can be used as prokinetic agents that alleviate gastrointestinal dysmotility [7]. Beyond antibiotic and prokinetic effects, macrolides can profoundly modulate the immune response in a variety of ways. Their immunomodulatory efficacy is well established when used chronically for respiratory diseases such as diffuse panbronchiolitis, chronic obstructive pulmonary disease and cystic fibrosis [8].

Rather than targeting a single disease mechanism, macrolides affect a multitude of immune receptors and pathways disturbed in sepsis, but their efficacy in this context has not been demonstrated unambiguously [9]. Animal studies have shown reduced inflammation, tissue damage and mortality, even in infections with macrolide-resistant bacteria [10–17]. Clinical studies hint that macrolide treatment may reduce mortality and the duration of symptoms in the most severely ill patients [18–22], and our group previously reported lower 30-day mortality in patients with acute respiratory distress syndrome (ARDS) treated with low-dose erythromycin [23]. In a study that assessed long-term outcomes and cost-effectiveness of clarithromycin treatment in patients with sepsis due to ventilator-associated pneumonia—conducted after a small RCT that showed no effect on overall 28-day mortality [18]—Tsaganos et al*.* reported a striking reduction in 90-day mortality in trial participants that received clarithromycin [20].

A potential long-term mortality benefit with macrolide treatment in sepsis has not been further substantiated in a randomized study design. Furthermore, no studies have explored the impact of immunomodulation by macrolides on the host response in patients with sepsis. We hypothesized that erythromycin improves clinical outcomes in sepsis by modulating underlying disease pathophysiology. By using prospective observational data to emulate a pragmatic RCT, we here aimed to estimate the effect of treatment with low-dose erythromycin (administered as a prokinetic agent) on mortality rate up to day 90 and the host response in critically ill patients with sepsis.

## Methods

### The MARS cohort

The MARS study (Molecular Assessment and Risk Stratification in Sepsis; ClinicalTrials.gov Identifier: NCT01905033) was a prospective cohort study conducted between January 2011 and December 2013 in two tertiary academic center adult ICUs in the Netherlands (Amsterdam University Medical Center, location AMC, and University Medical Center Utrecht). All admitted patients with an expected length of stay greater than 24 h were included via an opt-out consent procedure approved by both institutional medical ethics committees (IRB no. 10-056C). A more extensive description of this cohort can be found in Additional file 1: Methods and prior publications from our group [24–26].

### Clinical variables and definitions

Sepsis was defined as infection with a likelihood of possible, probable or definite diagnosed within 24 h after ICU admission [27, 28], and a modified sequential organ failure assessment (mSOFA) score (excluding the central nervous system component) of two or higher, consistent with sepsis-3 criteria [1]. In case of a missing SOFA score on admission, the presence of acute kidney injury (AKI) or ARDS upon ICU admission was considered a surrogate for a SOFA score of 2 or higher, thereby indicating eligibility for the study (3/705 [0.4%] patients in the final cohort). Other definitions can be found in Additional file 1: Methods.

All individual medication administrations during the study period were prospectively registered in MetaVision (iMDsoft, Israel). From these data, we identified whether patients received erythromycin (and other macrolides) and calculated the total administered dose, the duration of treatment and the total number of courses. We defined a new course of low-dose erythromycin as starting erythromycin again after at least 48 h of not receiving erythromycin.

### Study design, patient selection and outcomes

We designed this observational cohort study as a “target trial,” an emulation of the ideal RCT that could be used to answer the causal question of interest, within the constraints of the available data [29]. Explicitly specifying the study design this manner theoretically reduces the influence of biases common in non-randomized studies of interventions [29–31]. Additional file 1: Table 1 provides a side-by-side comparison of the target trial and its emulation described herein.

**Fig. 1:**
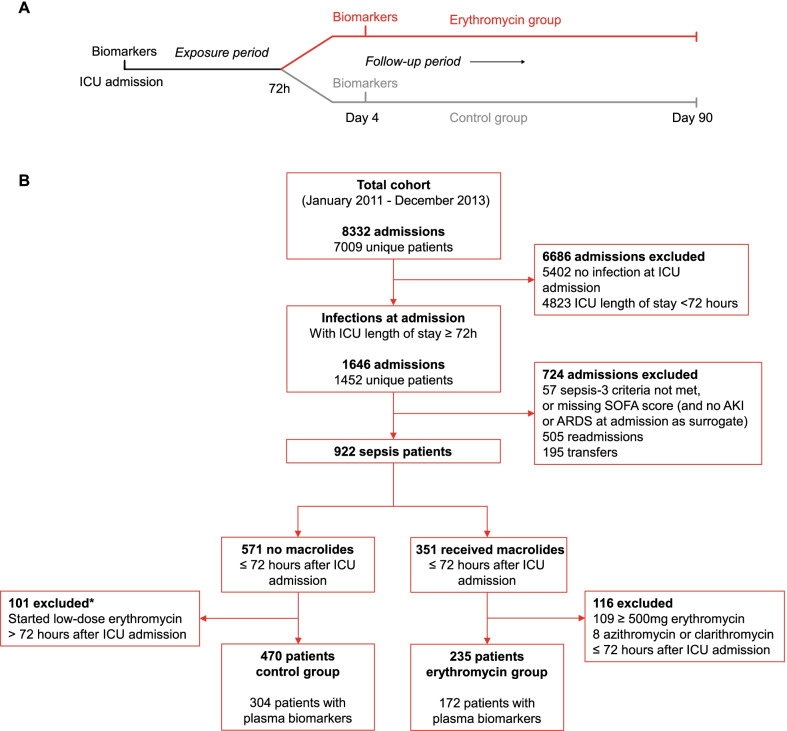
Study design and flowchart of patient selection. **A** Schematic representation of study design. Patients had to be alive and in the ICU during the first 72 h and were subsequently assigned to the erythromycin group or control group depending on whether they received low-dose erythromycin during these 72 h. The follow-up started after 72 h and lasted until day 90 after ICU admission. **B** Flowchart of patient selection. *These 101 patients were included in a sensitivity analysis. *ICU* intensive care unit

Patients were eligible for inclusion in this study if they met the criteria for sepsis within 24 h of ICU admission. Patients were ineligible if they had been readmitted following a previous ICU admission within the study period or if they were transferred from another hospital (unless this was on the first day of ICU admission). Figure 1A depicts the study design. All patients had to be alive and in the ICU during an exposure period of 72 h after ICU admission to prevent immortal time bias [32]. Patients were assigned to the erythromycin group if they had received erythromycin at least once at a low-dose (125–250 mg) within these 72 h or to the control group if they had not. The follow-up period started after this 72-h exposure period and ended 90 days after ICU admission.

The treatment strategy of interest was low-dose erythromycin (up to 600 mg per day, divided over 2–4 doses), administered as a prokinetic agent (i.e., to alleviate gastrointestinal dysmotility) during the first 72 h in ICU. We chose this indication to minimize the antimicrobial effects of erythromycin and consequently increase the likelihood that any remaining difference between groups could be attributed to its immunomodulatory effects. Previous studies on macrolide treatment in acute inflammation have focused more on azithromycin and clarithromycin prescribed at higher doses as antibiotics—often inferring immunomodulatory benefits from improvements in clinical outcomes despite causative microorganisms being macrolide-resistant [18, 19, 22]. We focused on low-dose erythromycin to study immunomodulatory macrolides in critically ill patients with sepsis for several reasons: our group previously demonstrated reduced 30-day mortality in patients with ARDS treated with low-dose erythromycin [23]; erythromycin improves outcomes in animal models relevant to sepsis [15–17]; while subtle differences have been reported, the effects of immunomodulatory macrolides are highly comparable and erythromycin, clarithromycin and azithromycin can often be used interchangeably [8, 33]; and the immunomodulatory effects of macrolides, at least in chronic use, occur at lower doses than the antimicrobial effects [8, 34, 35]. Azithromycin and clarithromycin were also occasionally administered in the participating ICUs during the study period, but we excluded patients using these drugs during the exposure period (*n* = 8), because azithromycin and clarithromycin were not used for the same indication, and together represented only 4.4% of the total individual macrolide administrations during the study period (523 out of 11,797).

Patients were excluded if they started high-dose erythromycin (500–1000 mg per administration), azithromycin or clarithromycin during the 72-h exposure period. In the per protocol analysis patients in the control group were excluded if they started low-dose erythromycin after 72 h during the same ICU admission, but these patients were included in an intention-to-treat sensitivity analysis. We did not exclude patients from either group who started high-dose erythromycin, azithromycin or clarithromycin after 72 h as part of their normal care.

The primary outcome was mortality rate up to 90 days. 30-day mortality was a secondary outcome. Secondary clinical outcomes indicative of the duration of symptoms were change in mSOFA score from admission until day 4 (“ΔSOFA”); ICU and hospital length of stay; and duration of mechanical ventilation. Secondary clinical outcomes indicative of ICU-acquired complications (occurring ≥ 72 h after ICU admission, after the exposure period) were the incidence of secondary infections, AKI and ARDS.

### Host response biomarker assays

Host response biomarkers were measured at admission (within 16 h of presentation) and day 4 in all sepsis patients of the MARS cohort with a likelihood of probable or definite enrolled during the first 2.5 years, as previously described [36]. Additional information pertaining to these measurements is provided in Additional file 1: Methods and Additional file 1: Table 2.

### Statistical methods

Categorical data are presented as count (percentage), normally distributed or non-normally distributed continuous data are presented as mean (standard deviation) or median [interquartile range], respectively. Baseline variables in the unadjusted table were compared between the erythromycin and control group using either Welch’s *t*-test or Wilcoxon’s rank-sum test (for normally or non-normally distributed continuous variables, respectively), or Fisher’s exact test (for categorical data). Tests were two-sided throughout, and a *P* value < 0.05 was considered statistically significant. All analyses were performed in the R statistical framework (version 4.1.2, Vienna, Austria).

### Missing data

Variables with ≤ 5% overall missing data were considered missing completely at random and hence were not imputed. The fraction of missing information for the covariates used in the propensity score (PS) estimation (listed below) was low, with values in any of the covariates missing in 18/705 (2.6%) patients. We therefore used a listwise deletion approach in which 5/235 (2.1%) patients in the erythromycin group and 13/470 (2.8%) patients in the control group were excluded from the analyses (Additional file 1: Fig. 1). A detailed overview of missing data is provided in Additional file 1: Methods and Additional file 1: Tables 3 and 4.

### Estimation of the propensity score

Treatment with erythromycin is dependent on baseline covariates linked to mortality. To deal with this confounding by indication (and thereby increase the likelihood of ignorability [37]), we used both PS matching and inverse probability of treatment weighting (IPTW; referred to as “weighting” or “weighted” throughout the manuscript) using the PS. These methods are commonly used to estimate different treatment effects: PS matching estimates the average treatment effect for the treated (ATT), the effect of the treatment for patients similar to those already being treated; IPTW estimates the average treatment effect (ATE), the effect of treatment if it were applied to the entire population under study [38].

For each patient, we estimated the PS—the probability of receiving the treatment given the covariates used in the model [39]—using logistic regression, with treatment exposure set as the dependent variable and baseline covariates as independent variables. Based on the pathophysiology of gastrointestinal dysmotility in the critically ill [7, 40, 41], we selected covariates either related to both receiving erythromycin and the primary outcome of 90-day mortality (true confounders), or related to 90-day mortality and possibly to erythromycin exposure (potential confounders). Variables only related to receiving erythromycin but not to the outcome were not included in the model [42]. Additional file 1: Fig. 2 depicts a directed acyclic graph (DAG) of the assumed causal relationships between the treatment, the outcome and the baseline (admission) covariates. The model included the following covariates (measured at ICU admission): age, sex, body mass index, hospital of admission, postsurgical admission, source of infection (abdominal, pulmonary, urinary, cardiovascular, skin, central nervous system or other/unknown [24]), Charlson comorbidity index score (without age), any malignancy (solid or hematological), Acute Physiology And Chronic Health Evaluation (APACHE) IV score [43], mSOFA score, Gastrointestinal Failure score—an ordinal scale ranging from “normal gastrointestinal function” to “abdominal compartment syndrome” [44]—dichotomized to absent (score of 0) or present (score of 1 or higher), septic shock, ARDS, AKI and use of mechanical ventilation.

For PS matching, we used greedy matching with a caliper width of 0.2 times the standard deviation of the PS logit [45] and matched treated patients to controls 1:1. For IPTW, we capped weights above 10 at 10 to limit excessive influence on the results induced by extremes of the PS. We assessed the balance in distribution of covariates before and after both PS matching and weighting by examining the standardized mean difference (SMD) for all variables, and the distribution of variances or interquartile ranges for continuous variables. SMDs should ideally be < 0.1 for all covariates used in the model, but we accepted SMDs up to 0.2. Variance ratios should ideally be 1, but values < 2 were considered acceptable [46].

### Estimation of the treatment effects

In the unadjusted sample, we compared mortality up to 90 days with Kaplan–Meier curves and estimated hazard ratios for mortality using Cox proportional hazard models. After PS matching, we created survival curves of the matched samples and estimated the hazard ratios and their standard errors by using Cox models with a robust variance estimator to account for the matched pairs [38]. After PS weighting, we created weighted survival curves and estimated hazard ratios using Cox models. We calculated the standard errors for the weighted hazard ratios as the standard deviation of the distribution of bootstrapped hazard ratios, specifically by re-estimating the weights and fitting the Cox model in 1000 bootstrap samples [47]. To assess the influence of residual confounding, we calculated E-values [48] as described in Additional file 1: Methods. Based on an earlier report [20], we also calculated hazard ratios for the period from 30 to 90 days after admission in the matched and weighted populations. Secondary clinical outcomes were compared using statistical tests appropriate for matched and weighted data, as described in Additional file 1: Methods.

### Analysis of host response biomarkers

We analyzed host response biomarker levels and trajectories in PS matched patients, using linear mixed models on log2-transformed values, as described in Additional file 1: Methods.

### Sensitivity analyses

We performed three sensitivity analyses to test whether findings in the primary outcome were robust to changes in the study design: (1) an intention-to-treat analysis where control patients in whom low-dose erythromycin was initiated more than 72 h after ICU admission were included (as excluding patients based on events that occur after follow-up has started may lead to selection bias); (2) an analysis with different exposure periods, in which we varied the duration of the period during which patients could be included (and had to be alive) from 72 to 48 h or 96 h; (3) a competing risk analysis in which we considered ICU discharge as a competing risk for mortality (see Additional file 1: Methods for details).

## Results

### Study population

Out of 8332 ICU admissions included in the MARS study between January 2011 and December 2013, 922 (11.1%) were first admission, non-transferred patients admitted with sepsis who stayed in the ICU for at least 72 h (Fig. 1B). Of these patients, 235 (25.5%) received low-dose erythromycin within 72 h of ICU admission. The potential control group consisted of 571 patients (61.9%) that did not receive any macrolides within the first 72 h. After excluding 101 patients from this group (17.7%) in whom low-dose erythromycin was initiated *after* the first 72 h, the control group consisted of 470 patients (Fig. 1).

Patients who received erythromycin were more often male (Table 1). While comorbidities and chronic medication associated with gastrointestinal dysmotility did not differ significantly between groups, other associated factors including postsurgical admission, higher disease severity (indicated by APACHE IV, SOFA scores and the presence of septic shock) and use of mechanical ventilation were more frequent in the erythromycin group. Consistent with erythromycin being prescribed as a prokinetic agent, the admission Gastrointestinal Failure score was higher in the erythromycin group, although this difference appeared mostly driven by the lower levels of the scale.

**Table 1:** Baseline characteristics and macrolide use

	Erythromycin (*n* = 235)	Controls (*n* = 470)	*P-*value	SMD*
Demographics				
Age, years	60.8 (13.6)	60.9 (15.3)	0.98	0.002
Sex, male	162 (68.9)	285 (60.6)	0.031	0.174
Body mass index, kg m^−2^	25.5 [23.0, 29.3]	25.5 [22.9, 29.1]	0.63	0.062
Race, white	208 (88.9)	417 (88.9)	> 0.99	0.001
Admission data				
Hospital A	149 (63.4)	233 (49.6)	0.001	0.282
Admission type, surgical	75 (31.9)	114 (24.3)	0.038	0.171
SDD use during admission^†^	181 (77.0)	325 (69.1)	0.033	0.178
Chronic comorbidities				
Charlson Comorbidity Index (without age)	2 [0, 4]	2 [0, 3]	0.31	0.066
Any malignancy	58 (24.7)	103 (21.9)	0.45	0.065
Non-metastatic solid tumor	33 (14.0)	49 (10.4)	0.17	0.111
Metastatic malignancy	6 (2.6)	19 (4.0)	0.39	0.083
Hematologic malignancy	23 (9.8)	37 (7.9)	0.39	0.068
Diabetes mellitus (type 1 or type 2)	49 (20.9)	100 (21.3)	0.92	0.010
Cerebrovascular disease	28 (11.9)	45 (9.6)	0.36	0.076
Hemiplegia	6 (2.6)	17 (3.6)	0.51	0.062
Chronic medication				
Any immunosuppressant	32 (13.7)	72 (15.8)	0.50	0.061
Antiplatelet drugs	63 (26.9)	123 (27.0)	> 0.99	0.002
Calcium-entry blockers	43 (18.3)	80 (17.1)	0.68	0.033
Beta-adrenergic blockers	65 (27.7)	129 (27.5)	> 0.99	0.003
Oral antidiabetic drugs	29 (12.3)	65 (13.9)	0.64	0.045
Insulin	26 (11.1)	52 (11.1)	> 0.99	0.001
Disease severity at ICU admission				
APACHE IV score	90.9 (28.5)	85.0 (28.4)	0.010	0.207
Acute physiology score	78.2 (26.4)	72.1 (26.4)	0.004	0.231
mSOFA score	8 [6, 11]	7 [5, 9]	< 0.001	0.455
Shock	176 (75.5)	242 (51.6)	< 0.001	0.514
ARDS	78 (33.2)	132 (28.1)	0.16	0.111
Mechanical ventilation	225 (96.6)	419 (89.3)	0.001	0.285
PaO_2_/FiO_2_ ratio^‡^	148 [99, 230]	156 [108, 217]	0.49	0.052
AKI	117 (49.8)	187 (39.8)	0.012	0.202
Gastrointestinal failure score			< 0.001	0.456
0—Normal gastrointestinal function	86 (36.6)	269 (57.2)		
1—Reduced/delayed enteral feeding^§^	112 (47.7)	133 (28.3)		
2—Food intolerance or IAH	35 (14.9)	65 (13.8)		
3—Food intolerance and IAH	2 (0.9)	2 (0.4)		
4—Abdominal compartment syndrome	0 (0.0)	0 (0.0)		
Gastrointestinal failure score > = 1	149 (63.4)	200 (42.6)	< 0.001	0.425
Gastrointestinal bleeding	7 (3.0)	8 (1.7)	0.28	0.085
Source of infection				
Pulmonary tract	119 (50.6)	252 (53.6)	0.47	0.060
Abdominal tract	48 (20.4)	83 (17.7)	0.41	0.070
Urinary tract	20 (8.5)	26 (5.5)	0.15	0.117
Cardiovascular	15 (6.4)	16 (3.4)	0.08	0.138
Skin	17 (7.2)	16 (3.4)	0.036	0.171
Central nervous system	6 (2.6)	29 (6.2)	0.042	0.178
Other or unknown	30 (12.8)	75 (16.0)	0.31	0.091
Erythromycin use				
First administration from admission, hours	38 [25, 52]			
Duration of the first course, hours	42 [24, 69]			
No. of administrations (first course)	5 [3, 8]			
Median dose per administration (first course), mg	200 [125, 200]			
Cumulative dose (first course), mg	800 [600, 1400]			
No. of courses during ICU stay				
1	182 (77.4)			
2	46 (19.6)			
3+	7 (3.0)			
Other macrolides				
High-dose erythromycin > 72 h after admission	0 (0.0)	1 (0.2)		
Azithromycin or clarithromycin > 72 h after admission	1 (0.4)	2 (0.4)		

*AKI* acute kidney injury, *APACHE-IV* acute physiology and chronic health evaluation IV, *ARDS* acute respiratory distress syndrome, *IAH* intraabdominal hypertension, *ICU* intensive care unit, *mSOFA* modified sequential organ failure assessment score (without the central nervous system component), *SDD* selective decontamination of the digestive tract, *SMD* standardized mean difference

^*^SMD > 0.2 indicates a substantial imbalance between groups; < 0.1 indicates a negligible difference

^†^Patients who did not receive SDD received selective oropharyngeal decontamination as part of a clinical trial [64]

^‡^Missing in 10/235 (4.3%) in the erythromycin group and 50/470 (10.6%) in the control group, see Additional file 1: Methods and Additional file 1: Table 3 for details

^§^“Enteral feeding < 50% of calculated needs or no feeding 3 days after abdominal surgery” in the original paper [44]

Categorical data are displayed as count (percentage) and compared using Fisher's exact test

Normally distributed continuous data are displayed as mean (standard deviation) and compared using Welch's *t*-test

Non-normally distributed continuous data are displayed as median [interquartile range] and compared using Wilcoxon's rank-sum test

Patients received their first dose of erythromycin at a median of 38 h (IQR 25–52 h) after ICU admission, and the total dose of this first course was low (median cumulative dose 800 mg [IQR 600-1400 mg] divided over a median of 5 [IQR 3–8] total administrations; Table 1). A negligible proportion of patients received a macrolide as an antibiotic more than 72 h after ICU admission (1/235, 0.4%, in the erythromycin group, 3/470, 0.6%, in the control group).

### Clinical outcomes

**Fig. 2:**
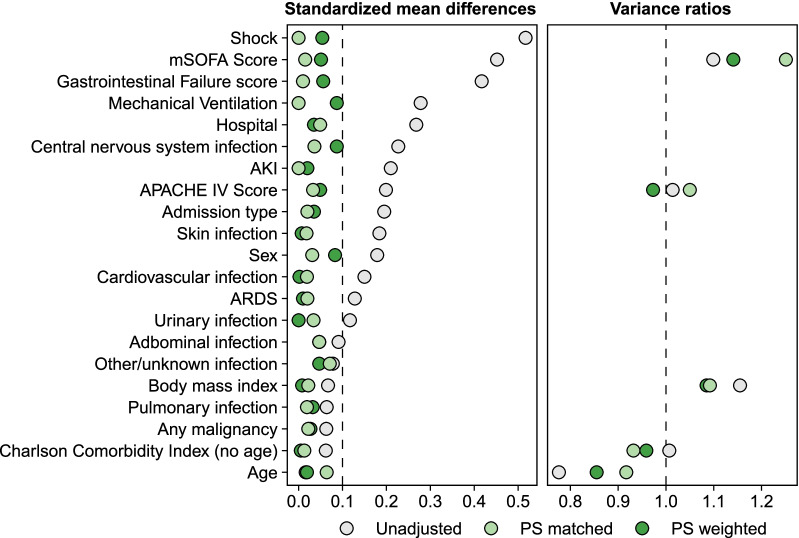
Balance statistics of the covariates used for PS matching and weighting. Plot depicting the (absolute) SMDs and the variance ratios between the unadjusted and the PS matched or PS weighted populations for the covariates used in the model to estimate the propensity scores. The unadjusted SMDs were obtained prior to PS weighting. SMDs should ideally be < 0.1 (left dashed vertical line); variance ratios should ideally be 1 (right dashed vertical line), but between 0.5 and 2 is acceptable. *AKI* acute kidney injury, *APACHE-IV* acute physiology and chronic health evaluation IV, *ARDS* acute respiratory distress syndrome, *mSOFA* modified sequential organ failure assessment score (without the central nervous system component), *PS* propensity score, *SMD* standardized mean difference

After excluding 5 patients in the erythromycin group (2.1%) and 13 patients in the control group (2.8%) because of missing data (see Additional file 1: Methods, Additional file 1: Table 3 and Additional file 1: Fig. 1 for details), 230 and 457 patients were available for matching and weighting. Both matching and weighting resulted in balanced distribution of the covariates used in the model and covariates not used in the model (Fig. 2 and Additional file 1: Table 5).

**Fig. 3:**
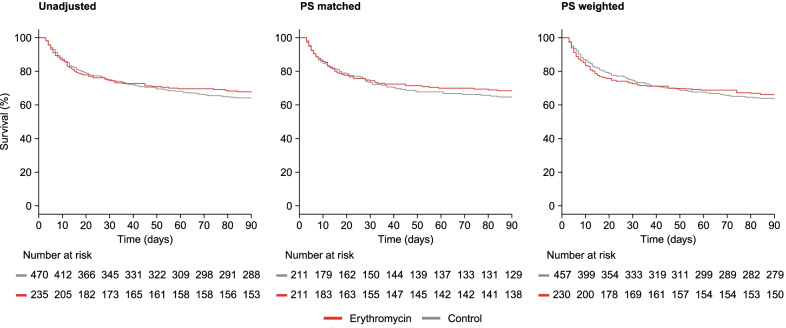
Kaplan–Meier survival curves for the unadjusted population, and the PS matched and weighted populations. The number at risk for the PS weighted population indicate the numbers of patients with complete data included in this analysis (the individual weights are not applied to these numbers). *PS* propensity score

After matching and weighting, we found no differences in mortality rate up to 90 days: matching HR 0.89 (95% CI 0.64–1.24), weighting HR 0.95 (95% CI 0.66–1.36; Fig. 3 and Table 2). The E-Values for shifting these hazard ratios to a range consistent with either benefit (upper limit of the 95% CI to < 1.00) or harm (lower limit of the 95% CI to > 1.00) ranged from 1.61 to 2.08 (Additional file 1: Table 6), which makes it unlikely that (unmeasured) residual confounding would result in evidence of benefit or harm (see Additional file 1). In addition, we found no evidence for time-varying differences in mortality between groups (matching HR_30-90 days_ 0.69 [95% CI 0.34–1.41]; weighting HR_30-90 days_ 0.59 [95% CI 0.28–1.28]). Similarly, 30-day mortality was not different between groups (Table 3).

**Table 2:** Hazard ratios for mortality up to day 90

	90-day mortality rate	
Unadjusted	Events, *n* (%)	HR (95% CI)
Erythromycin (n = 235)	76 (33.3%)	0.91 (0.69–1.19)
Controls (n = 470)	167 (36.8%)	1.00 (ref)

PS matched		
Erythromycin (n = 211)	67 (32.8%)	0.89 (0.64–1.24)
Controls (n = 211)	74 (36.6%)	1.00 (ref)

PS weighted	Events, %	
Erythromycin	34.6%	0.95 (0.66–1.36)
Controls	37.3%	1.00 (ref)

*CI* confidence interval, *HR* hazard ratio, *IPTW* inverse probability of treatment weighting, *PS* propensity score, *ref* referent

**Table 3:** Secondary clinical outcomes

	Unadjusted			PS matched			PS weighted		
	Erythromycin (*n* = 235)	Controls (*n* = 470)	*P *value	Erythromycin (*n* = 211)	Controls (*n* = 211)	*P *value	Erythromycin	Controls	*P *value
30-day mortality	60 (25.9)	117 (25.4)	0.93	54 (26.0)	56 (27.3)	0.91	27.7	25.9	0.67
ICU length of stay, days	8 [5, 14]	7 [4, 11]	< 0.001	8 [5, 13]	8 [5, 13]	0.51	7 [5, 13]	7 [4, 11]	0.06
Hospital length of stay, days	23 [13, 40]	19 [11, 36]	0.031	22 [12, 37.5]	21 [12, 39]	0.61	21 [10, 37]	20 [11, 37]	0.69
Duration of mechanical ventilation, days*	6 [4, 11]	5 [3, 9]	0.001	6 [4, 11]	6 [3, 10]	0.48	6 [4, 10]	5 [3, 9]	0.11
ΔSOFA day 4^†^	−1.5 (2.9)	−1.2 (2.6)	0.35	−1.5 (2.9)	−1.6 (2.7)	0.79	−1.3 (2.8)	−1.4 (2.7)	0.75
Incidence of ICU-acquired infections	42 (17.9)	51 (10.9)	0.013	35 (16.6)	32 (15.2)	0.79	13.8	12.0	0.51
Incidence of ICU-acquired AKI	17 (7.2)	24 (5.1)	0.31	16 (7.6)	15 (7.1)	> 0.99	6.4	5.5	0.66
Incidence of ICU-acquired ARDS	9 (3.8)	19 (4.0)	> 0.99	8 (3.8)	13 (6.2)	0.38	3.3	4.6	0.43

*AKI* acute kidney injury, *ARDS* acute respiratory distress syndrome, *ICU* intensive care unit, *PS* propensity score, *ΔSOFA* change in modified sequential organ failure assessment score (excluding the neurological component) from admission to day 2, 3 or 4

Categorical data are displayed as count (percentage) or and compared using Fisher's exact test (unadjusted) or McNemar's test (after PS matching), or displayed as percentage and compared using a Chi-square test^‡^ (after PS weighting)

Normally distributed continuous data are displayed as mean (standard deviation) and compared using a *t*-test (unadjusted), a paired *t*-test (after PS matching) or a *t*-test^‡^ (after PS weighting)

Non-normally distributed continuous data are displayed as median [interquartile range] and compared using Wilcoxon's rank-sum test (unadjusted), Wilcoxon's signed-rank test or Wilcoxon rank-sum test^‡^ (after PS weighting)

^*^In those who were mechanically ventilated at ICU admission: 225/235 (96.6%) in the erythromycin group, 419/470 (89.3%) in the control group (in the unadjusted population)

^†^Missing in 21/235 (8.9%) in the erythromycin group and 102/470 (21.7%) in the control group, see Additional file 1: Methods and Additional file 1: Table 3 for details

^‡^For weighted samples, as provided in the *survey* R package

In the unadjusted cohort, patients who received erythromycin had longer ICU- and hospital lengths of stay, duration of mechanical ventilation and more frequently developed ICU-acquired infections. However, none of these differences remained after matching and weighting (Table 3).

### Host response biomarkers

**Fig. 4:**
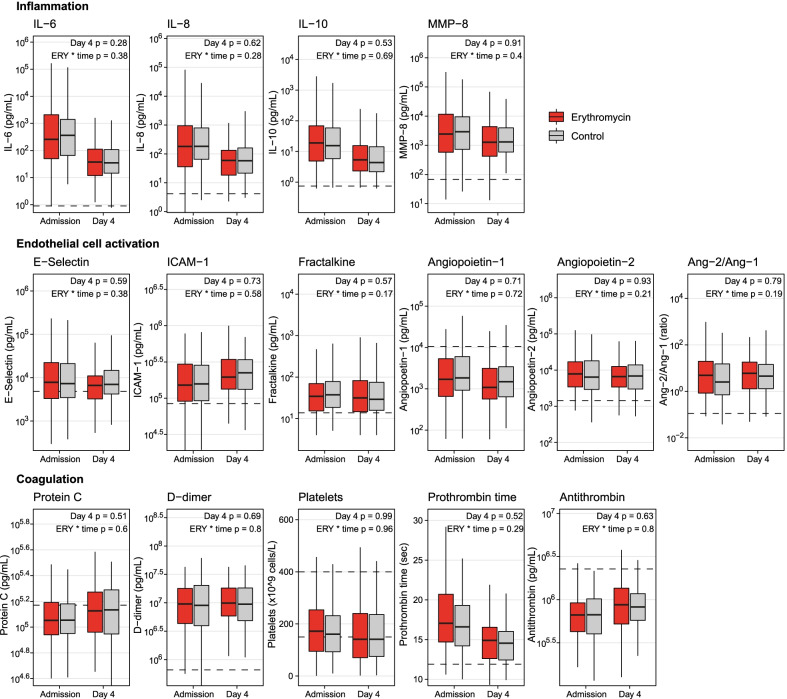
Host response biomarkers reflecting inflammation, endothelial cell activation and coagulation in PS matched treated (*n* = 150) and control (*n* = 150) patients. The box represents the 25th percentile, median and 75th percentile. The whiskers represent up to 1.5 times the interquartile range. The dashed line represents the median value in healthy volunteers or the normal range in the reference clinical laboratory (for prothrombin time and platelets). The *P* values are derived from linear mixed models using log2-transformed biomarkers as the dependent variable and including a random slope and intercept for the change over time per patient. “Day 4 p” is the difference between groups at day 4 (obtained as the *P* value for the treatment coefficient in models using day 4, rather than admission, as the reference category). “ERY * time p” is the interaction term for treatment and time, i.e., whether the slope over time (from admission to day 4) differs between groups. *ERY* erythromycin

We next sought to ascertain levels of key biomarkers reflective of the septic host response in the domains of inflammation, endothelial cell activation and coagulation, before and after 4 days in erythromycin-treated and control patients. PS matching of patients with complete data in whom host response biomarkers were measured—170 in the erythromycin group and 295 in the control group (Additional file 1: Fig. 1)—resulted in a balanced population of 150 1:1 matched patient pairs (see Additional file 1: Table 7 for baseline characteristics, Additional file 1: Fig. 3 for balancing statistics). This balance was also reflected in the admission biomarker levels (prior to treatment), which were all comparable between treated and control patients. When assessing biomarker levels at day 4, and the change from admission to day 4, we found no differences between patients treated with erythromycin and controls on any of the measured host response biomarkers (Fig. 4; Additional file 1: Fig. 4 for IL-8/IL-10 ratio and IL-6/IL-10 ratio).

### Sensitivity analyses

Allowing the 101 patients that received erythromycin more than 72 h *after* ICU admission to be in the control group led to a cohort of 235 treated patients and 571 controls (baseline characteristics in Additional file 1: Table 8). After matching and weighting (Additional file 1: Table 9 for matched and weighted populations, and Additional file 1: Fig. 5 for balancing statistics), we found no significant differences in mortality rate (Additional file 1: Table 10), nor for the secondary clinical outcomes (Additional file 1: Table 11).

Altering the exposure period during which patients had to be alive and could be assigned to the treatment or control groups from 72 h to 48 or 96 h—and once again excluding control patients in whom erythromycin was started after this exposure period—resulted in cohorts of 191 treated and 637 control patients for the 48-h exposure period, and 242 treated and 371 control patients for the 96-h exposure period (Additional file 1: Tables 12 and 13). Despite these substantial shifts in group numbers, after matching and weighting (Additional file 1: Tables 14 and 15, Additional file 1: Figs. 6 and 7) the conclusions remained unchanged: we found no differences in mortality rate up to day 90 between groups (Additional file 1: Tables 16 and 17), nor in most secondary clinical outcomes (with the exception of a slightly longer ICU length of stay and duration of mechanical ventilation in the erythromycin group with the 48-h exposure period; Additional file 1: Tables 18 and 19). Finally, time to event analyses in which we considered ICU discharge as a competing risk for mortality did not result in differences between groups after PS matching (cause-specific HR for mortality 0.97 [0.65–1.46], subdistribution HR for mortality 0.96 [0.64–1.43]; Additional file 1: Table 20).

## Discussion

Using observational data to emulate a target trial, we here aimed to assess the effect of treatment with low-dose erythromycin on the outcome of critically ill patients with sepsis—a syndrome with major global impact but no targeted treatment options to date [2, 5]. We could not demonstrate an effect of low-dose erythromycin on 90-day mortality, nor on secondary outcomes indicative of duration of symptoms, occurrence of ICU-acquired complications or levels of biomarkers reflective of the septic host response. These results, while perhaps limited by the low total dose and short duration of erythromycin treatment, do not argue in favor of using low-dose erythromycin as an adjunctive immunomodulatory therapy in this population, although more studies are needed to obtain more precise effect estimates.

Macrolides exert an array of immunomodulatory and other non-antibiotic effects in vitro and in vivo that could, at least in theory, benefit critically ill patients, including those with sepsis [9]. We chose primary and secondary surrogate or patient-important outcomes that could reflect these effects. Macrolides may reduce excessive inflammation and thereby prevent organ damage (including ventilator-induced lung injury) and expedite the return to immune homeostasis [13, 49–51]; we did not find an effect on secondary outcomes indicative of duration of symptoms, incidence of inflammation-associated complications (AKI, ARDS) or biomarkers reflective of inflammation. Macrolides may stimulate key host immune defenses—including phagocytosis and intracellular killing, commonly impaired in sepsis-induced immune suppression [52]—and interfere with microbial virulence mechanisms such as biofilm formation [53–55]; we did not find a reduction in ICU-acquired secondary infections. Ultimately, we expected that the synergistic effect of these processes could, as it does in animal models [10–14], reduce mortality rates for critically ill patients with sepsis in the both short term (by preventing organ failure) and longer term (by preventing secondary infections); we did, however, not find an effect on mortality.

Previous clinical studies that investigated the immunomodulatory effects of macrolides in critically ill patients, while limited in number, both corroborate and contrast the findings presented here. Most published observational studies, both in sepsis and ARDS (often caused by sepsis and exhibiting similar immune disturbances), have reported lower mortality rates and reduced duration of symptoms in patients treated with macrolides [21–23, 56–58]. Two RCTs have been published, in which patients with sepsis due to microorganisms likely to be macrolide-resistant received clarithromycin (in antibiotic doses, 1 g once per day for three days [18] or four days [19]). The first trial, in 200 patients with sepsis due to ventilator-associated pneumonia, reported no reduction in 28-day mortality [18], but a remarkable reduction in 90-day mortality in a follow-up study [20]. The second trial, in 600 patients with sepsis likely due to gram-negative bacteria, similarly did not find an effect on 28-day mortality (to the best of our knowledge, 90-day mortality data are not available for this trial [19]). In secondary analyses, both trials did present results consistent with a mortality benefit for clarithromycin in the most severely ill patients (those with septic shock and multiple organ dysfunction syndrome) and a reduction in duration of symptoms. While the results of our study regarding 30-day mortality are in line with these two trials, discrepancies in other outcomes may be explained by several factors: (1) a different drug, as different immunomodulatory macrolides may exhibit subtle differences in effects [8]; (2) a lower dose in our study, for a briefer and more varied duration; (3) differences in the study population, such as different sources of infection; (4) the presence of gastrointestinal dysmotility, which could still affect patient prognosis in ways not captured by baseline covariates; or (5) a slightly different time window, as our study could, by design, only assess outcomes occurring more than 72 h after ICU admission. We eagerly await the results of a third trial (ClinicalTrials.gov Identifier: NCT03345992), which only included patients with multiple organ dysfunction syndrome, who were most likely to benefit from adjunctive macrolide treatment in the two previous trials (recruitment has concluded, but the results are not available at the time of writing).

We used a target trial emulation approach to reduce the influence of biases common to non-randomized studies of interventions [29–31]. This emulation is always performed within the constraints of the available An important deviation that our data made from the target trial designed to assess the immunomodulatory effectiveness of low-dose erythromycin is that, in a trial, treatment with erythromycin would not be limited to patients with gastrointestinal dysmotility. We chose this indication to infer immunomodulatory effects from the absence (or negligible presence) of antibacterial effects, but we cannot exclude an effect of reduced gastrointestinal dysmotility on the outcomes—which could, in theory, either oppose or augment the immunomodulatory effects. Not having to account for this indication would both eliminate residual confounding by indication and any post-baseline effects that gastrointestinal dysmotility would have on the mortality (e.g., nutritional deficiencies or intestinal bacterial translocation leading to new infections [41]). To illustrate this point: several observational studies have reported worse outcomes in patients with gastrointestinal dysmotility even after controlling for disease severity [41]. Nevertheless, we consider it unlikely that any (unmeasured) confounding variable would be strong enough to reject the null hypothesis (no difference between groups), as indicated by the E-Values [48] for the primary analyses described in Additional file 1.

Another deviation from the target trial pertains to the large between-patient variation in total dose and duration of erythromycin treatment, because the necessary total dose and duration to achieve sufficient immunomodulatory effects in acute critical illness are unknown. For most patients in the erythromycin group, the cumulative dose and duration of the first course were low: a median of 800 mg over a median of 42 h (divided over a median of 5 administrations), whereas an antibiotic dose would commonly be up to 2000 mg per day for several days. A per protocol treatment directly comparable with the two RCTs using clarithromycin [18, 19] would consist of 2000 mg per day for 72 or 96 h. Despite these considerations, the immunomodulatory effects of macrolides do occur at much lower doses than the antibiotic effects (e.g., 400–600 mg erythromycin per day for diffuse panbronchiolitis; 500 mg erythromycin twice daily for chronic obstructive pulmonary disease), although chronic use may be needed for some of these effects to occur [8, 34, 35].

Several strengths and limitations of this study—partly discussed in the preceding paragraphs—are worth emphasizing. Strengths include the target trial study design, the comprehensiveness of the available data, the use of a DAG to identify confounding covariates, and the robustness of the results to different analysis techniques (matching for the ATT, weighting for the ATE) and sensitivity analyses. Limitations include the aspects of study design that deviate from the target trial (e.g., indication of gastrointestinal dysmotility, uncertainty of the per protocol dose). Also, due to limitations of sample size, considerable statistical uncertainty remains in our effect estimates, making it impossible to exclude potentially meaningful benefits or harms of treatment. In addition, we cannot fully exclude prevalent user bias [59], because data on macrolide use prior to ICU admission were unavailable. We nevertheless considered this type of bias unlikely, as low-dose erythromycin is not commonly prescribed for adults in the Netherlands in outpatient, emergency department or hospital ward settings, and patients receiving clarithromycin or azithromycin upon ICU admission were excluded. Furthermore, only including patients who survive the first 72 h (to prevent immortal time bias) means our results cannot be generalized to patients who leave the ICU before this time window.

Future studies on immunomodulation by macrolides in acute inflammation and critical illness should assess the timing, dose and duration of treatment required to achieve immunomodulatory effects—as measured by plasma biomarkers, immune cell phenotype or function, or other indices—and subsequently assess whether this relates to clinical outcomes. A precision medicine approach, such as those based on clinical phenotypes or molecular endotypes [60, 61], may help separate patients who benefit from macrolide treatment from those in whom macrolides could be detrimental (e.g., patients with cardiovascular comorbidities [62]). Lastly, it may be of interest to study alternative clinical outcomes for which non-antibiotic benefits of macrolides are biologically plausible, such as the prevention of ICU-acquired infections (in particular ventilator-associated pneumonia [9, 63]).

## Conclusion

In this target trial emulation performed in a prospectively enrolled cohort of critically ill patients with sepsis, we could not demonstrate an effect of erythromycin on clinical outcomes and host response biomarkers. Despite noteworthy deviations from the target trial—in particular the variation in total dose and duration of erythromycin treatment—these results do not support the use of erythromycin for this purpose. Additional studies and meta-analyses are required to obtain more precise effect estimates.

## Supplementary Information

**Additional file 1.** Supplementary Information. Supplementary methods, tables and figures.

## Data Availability

The datasets used and/or analyzed during the current study are available from the corresponding author on reasonable request.

## Abbreviations

AKI: Acute kidney injury
APACHE IV: Acute physiology and chronic health evaluation IV
ARDS: Acute respiratory distress syndrome
ATE: Average treatment effect
ATT: Average treatment effect on the treated
CI: Confidence interval
DAG: Directed acyclic graph
ICU: Intensive care unit
HR: Hazard ratio
IPTW: Inverse probability of treatment weighting
PS: Propensity score
RCT: Randomized clinical trial
SMD: Standardized mean difference
SOFA: Sequential organ failure assessment score
