# Supplementary material for: Effect of erythromycin on mortality and the host response in critically ill patients with sepsis: a target trial emulation
Source: Crit Care. 2022 May 24;26:151. doi: 10.1186/s13054-022-04016-x (PMC9128233; doi:10.1186/s13054-022-04016-x)
Supplement: Supplementary file 1 — Additional file 1. Supplementary Information. Supplementary methods, tables and figures. [file 13054_2022_4016_MOESM1_ESM.docx]

**SUPPLEMENTARY INFORMATION**

**Effect of erythromycin on mortality and the host response in critically ill patients with sepsis: a target trial emulation**

Tom D.Y. Reijnders^1^*, Hessel Peters-Sengers^1^, Lonneke A. van Vught^1^, Fabrice Uhel^1-4^, Marc J.M. Bonten^5,6^ , Olaf L. Cremer^7^, Marcus J. Schultz^8-10^, Martijn M. Stuiver^11^, Tom van der Poll^1,12^, on behalf of the MARS consortium

^1^Center for Experimental and Molecular Medicine, Amsterdam University Medical Centers, location Academic Medical Center, Amsterdam, the Netherlands. ^2^AP-HP, Hôpital Louis Mourier, DMU ESPRIT, Médecine Intensive-Réanimation, F-92700, Colombes, France. ^3^Université de Paris, UFR de Médecine, F-75018, Paris, France. ^4^INSERM U1151, CNRS UMR 8253, Institut Necker-Enfants Malades, F-75015 Paris, France ^5^Department of Medical Microbiology, University Medical Center Utrecht, Utrecht, the Netherlands. ^6^Julius Center for Health Sciences and Primary Care, University Medical Center Utrecht, Utrecht, the Netherlands. ^7^Department of Intensive Care Medicine, University Medical Center Utrecht, Utrecht, the Netherlands. ^8^Departments of Intensive Care Medicine, and Laboratory of Experimental Intensive Care and Anesthesiology (LEICA), Amsterdam University Medical Centers, location Academic Medical Center, Amsterdam, The Netherlands. ^9^Mahidol-Oxford Tropical Medicine Research Unit (MORU), Mahidol University, Bangkok, Thailand. ^10^Nuffield Department of Medicine, University of Oxford, Oxford, United Kingdom. ^11^Department of Epidemiology and Data Science, Amsterdam Public Health, Amsterdam UMC, location Academic Medical Center, Amsterdam, The Netherlands. ^12^Division of Infectious Diseases, Amsterdam University Medical Centers, location Academic Medical Center, University of Amsterdam, Amsterdam, The Netherlands.

***Corresponding author:** Tom D.Y. Reijnders, Center for Experimental and Molecular Medicine, Academic Medical Center, Room T1.0-236. Meibergdreef 9, 1105 AZ Amsterdam, the Netherlands.

Telephone: +31 20 5664903. E-mail: [t.d.reijnders@amsterdamumc.nl](mailto:t.d.reijnders@amsterdamumc.nl)

**TABLE OF CONTENTS**

[TABLE OF CONTENTS 2](#_Toc100384012)

[STROBE STATEMENT 5](#_Toc100384013)

[SUPPLEMENTARY METHODS 7](#_Toc100384014)

[The MARS cohort 7](#_Toc100384015)

[Clinical variables and definitions 7](#_Toc100384016)

[Handling of missing baseline and outcome data 7](#_Toc100384017)

[Host response biomarker assays and handling of missing biomarker data 8](#_Toc100384018)

[Analysis of secondary clinical outcomes 9](#_Toc100384019)

[Analysis of host response biomarkers 9](#_Toc100384020)

[Calculation of E-Values to assess residual confounding 10](#_Toc100384021)

[Competing risk analysis 11](#_Toc100384022)

[R packages 11](#_Toc100384023)

[SUPPLEMENTARY TABLES 12](#_Toc100384024)

[Supplementary table 1. Study protocol for the target trial and its emulation 12](#_Toc100384025)

[Supplementary Table 2. Overview of host response biomarker measurements 13](#_Toc100384026)

[Supplementary Table 3. Overview of missing clinical data 14](#_Toc100384027)

[Supplementary Table 4. Overview of missing biomarker data 16](#_Toc100384028)

[Supplementary Table 5. PS matched and weighted populations for the primary analysis 17](#_Toc100384029)

[Supplementary Table 6. E-Values to shift hazard ratios of the primary analysis to a range consistent with harm or benefit 19](#_Toc100384030)

[Supplementary Table 7. Baseline characteristics and PS matched population for the host response biomarker analyses 20](#_Toc100384031)

[Supplementary Table 8. Baseline characteristics for the intention-to-treat sensitivity analysis 22](#_Toc100384032)

[Supplementary Table 9. PS matched and PS weighted populations for the intention-to-treat sensitivity analysis 24](#_Toc100384033)

[Supplementary Table 10. Hazard ratios for mortality up to day 90 for the intention-to-treat sensitivity analysis 26](#_Toc100384034)

[Supplementary Table 11. Secondary clinical outcomes for the intention-to-treat sensitivity analysis 27](#_Toc100384035)

[Supplementary Table 12. Baseline characteristics for the sensitivity analysis with an exposure period of 48 hours 28](#_Toc100384036)

[Supplementary Table 13. Baseline characteristics for the sensitivity analysis with an exposure period of 96 hours 30](#_Toc100384037)

[Supplementary Table 14. PS matched and PS weighted populations for the sensitivity analysis with an exposure period of 48 hours 32](#_Toc100384038)

[Supplementary Table 15. PS matched and PS weighted populations for the sensitivity analysis with an exposure period of 96 hours 34](#_Toc100384039)

[Supplementary Table 16. Hazard ratios for mortality up to day 90 for the sensitivity analysis with an exposure period of 48 hours 36](#_Toc100384040)

[Supplementary Table 17. Hazard ratios for mortality up to day 90 for the sensitivity analysis with a grace period of 96 hours 37](#_Toc100384041)

[Supplementary Table 18. Secondary clinical outcomes for the sensitivity analysis with a grace period of 48 hours 38](#_Toc100384042)

[Supplementary Table 19. Secondary clinical outcomes for the sensitivity analysis with a grace period of 96 hours 39](#_Toc100384043)

[Supplementary Table 20. Cause-specific and subdistribution hazard ratios for ICU discharge and mortality for treatment with erythromycin 40](#_Toc100384044)

[SUPPLEMENTARY FIGURES 41](#_Toc100384045)

[Supplementary Figure 1. Flowchart of patients used in estimating the PS and subsequent analyses (complete cases) 41](#_Toc100384046)

[Supplementary Figure 2. Proposed directed acyclic graph 42](#_Toc100384047)

[Supplementary Figure 3. Balance statistics of the covariates used for PS matching in the population with measured biomarkers 43](#_Toc100384048)

[Supplementary Figure 4. IL-8/IL-10 and IL-6/IL-10 ratios in PS matched treated (n = 150) and control (n = 150) patients 44](#_Toc100384049)

[Supplementary Figure 5. Balance statistics of the covariates used for PS matching and weighting for the intention-to-treat sensitivity analysis 45](#_Toc100384050)

[Supplementary Figure 6. Balance statistics of the covariates used for PS matching and weighting for the sensitivity analysis with an exposure period of 48 hours 46](#_Toc100384051)

[Supplementary Figure 7. Balance statistics of the covariates used for PS matching and weighting for the sensitivity analysis with an exposure period of 96 hours 47](#_Toc100384052)

[REFERENCES 48](#_Toc100384053)

# STROBE STATEMENT

|  | **Item** | **Recommendation** | **Page** |
| --- | --- | --- | --- |
| **Title and abstract** | 1 | (*a*) Indicate the study’s design with a commonly used term in the title or the abstract | 1 |
|  |  | (*b*) Provide in the abstract an informative and balanced summary of what was done and what was found | 2 |
| **Introduction** | | |  |
| Background/rationale | 2 | Explain the scientific background and rationale for the investigation being reported | 4-5 |
| Objectives | 3 | State specific objectives, including any prespecified hypotheses | 4-5 |
| **Methods** | | |  |
| Study design | 4 | Present key elements of study design early in the paper | 5-7 |
| Setting | 5 | Describe the setting, locations, and relevant dates, including periods of recruitment, exposure, follow-up, and data collection | 5-7, Sup. 6 |
| Participants | 6 | (*a*) Give the eligibility criteria, and the sources and methods of selection of participants. Describe methods of follow-up | 5-7, Sup. 6 |
|  |  | (*b*) For matched studies, give matching criteria and number of exposed and unexposed | 8-9 |
| Variables | 7 | Clearly define all outcomes, exposures, predictors, potential confounders, and effect modifiers. Give diagnostic criteria, if applicable | 5-9, Sup. 6, Sup. Fig. 2 |
| Data sources/ measurement | 8 | For each variable of interest, give sources of data and details of methods of assessment (measurement). Describe comparability of assessment methods if there is more than one group | 5-7, Sup. 6 |
| Bias | 9 | Describe any efforts to address potential sources of bias | 5-10 |
| Study size | 10 | Explain how the study size was arrived at | 10, Fig. 1 |
| Quantitative variables | 11 | Explain how quantitative variables were handled in the analyses. If applicable, describe which groupings were chosen and why | 7-10 |
| Statistical methods | 12 | (*a*) Describe all statistical methods, including those used to control for confounding | 7-10, Sup. 8-10 |
|  |  | (*b*) Describe any methods used to examine subgroups and interactions | N/A |
|  |  | (*c*) Explain how missing data were addressed | 8, Sup. 6-8 |
|  |  | (*d*) If applicable, explain how loss to follow-up was addressed | Sup. 7 |
|  |  | (*e*) Describe any sensitivity analyses | 10, Sup. 9-10 |
| **Results** | | |  |
| Participants | 13 | (a) Report numbers of individuals at each stage of study—eg numbers potentially eligible, examined for eligibility, confirmed eligible, included in the study, completing follow-up, and analysed | 10-12, Fig. 1, Sup. Fig. 1 |
|  |  | (b) Give reasons for non-participation at each stage | 10, Fig. 1 |
|  |  | (c) Consider use of a flow diagram | Fig. 1, Sup. Fig. 2 |
| Descriptive data | 14* | (a) Give characteristics of study participants (eg demographic, clinical, social) and information on exposures and potential confounders | 10, Table 1 |
|  |  | (b) Indicate number of participants with missing data for each variable of interest | 11, Sup. Tables 3-4 |
|  |  | (c) Summarise follow-up time (eg, average and total amount) |  |
| Outcome data | 15 | Report numbers of outcome events or summary measures over time | Tables 2-3 Fig. 3 |
| Main results | 16 | (*a*) Give unadjusted estimates and, if applicable, confounder-adjusted estimates and their precision (eg, 95% confidence interval). Make clear which confounders were adjusted for and why they were included | 8-9, 11, Table 2 |
|  |  | (*b*) Report category boundaries when continuous variables were categorized | N/A |
|  |  | (*c*) If relevant, consider translating estimates of relative risk into absolute risk for a meaningful time period | N/A |
| Other analyses | 17 | Report other analyses done—eg analyses of subgroups and interactions, and sensitivity analyses | 12-13, Sup. 8-10 |
| **Discussion** | | |  |
| Key results | 18 | Summarise key results with reference to study objectives | 13-16 |
| Limitations | 19 | Discuss limitations of the study, taking into account sources of potential bias or imprecision. Discuss both direction and magnitude of any potential bias | 15-16 |
| Interpretation | 20 | Give a cautious overall interpretation of results considering objectives, limitations, multiplicity of analyses, results from similar studies, and other relevant evidence | 13-16 |
| Generalisability | 21 | Discuss the generalisability (external validity) of the study results | 16 |
| **Other information** | | |  |
| Funding | 22 | Give the source of funding and the role of the funders for the present study and, if applicable, for the original study on which the present article is based | 17 |

**SUPPLEMENTARY METHODS**

***The MARS cohort***

The MARS study (Molecular Assessment and Risk Stratification in Sepsis; ClinicalTrials.gov Identifier: NCT01905033) was a prospective cohort study conducted between January 2011 and December 2013 in two tertiary academic center adult ICUs in the Netherlands (Amsterdam University Medical Center, location AMC, and University Medical Center Utrecht). All admitted patients with an expected length of stay greater than 24 hours were included via an opt-out consent procedure approved by both institutional medical ethics committees (IRB no. 10-056C). Clinical data were prospectively collected by trained physician researchers or obtained from the patient data management system (MetaVision, iMDsoft, Tel Aviv, Israel), and plasma remaining after routine laboratory measurements (that would otherwise have been discarded) was stored for future analyses. Patients with a clinically suspected infection were assessed post-hoc (by a team of physicians) to determine the likelihood of the infection according to the Centers for Disease Control and Prevention and International Sepsis Forum criteria: definite, probable, possible, or none [1]. Survival status up to one year was determined through the governmental personal records database. A more extensive description of this cohort can be found in prior publications from our group [2–4].

## Clinical variables and definitions

AKI was defined in accordance according to the RIFLE criteria (score of 1 or higher) [5]. The presence of ARDS was prospectively scored according to the American-European Consensus Criteria [6] (although all these patients also meet the more recent Berlin definition of ARDS) [7,8]. The presence of septic shock was defined as the use of vasopressors for hypotension in a dose of at least 0.1 μg/kg/min of norepinephrine (or equivalent doses of other vasopressors) during at least 50% of the day in the ICU. Chronic comorbidities were scored prospectively according to the Charlson Comorbidity Index [9].

***Handling of missing baseline and outcome data***

An overview missing baseline characteristics and outcomes with reason for missingness can be found in Supplementary Table 3. Variables with ≤ 5% overall missing data were assumed missing completely at random (MCAR) and therefore not imputed. All missing baseline characteristics that were used in estimating the propensity scores (PS; based on the directed acyclic graph shown in Supplementary Fig. 2) were considered MCAR. The fraction of missing information for the covariates used in estimating the propensity score was low, with missing values in any of the covariates in 18/705 (2.6%) patients. We therefore used a listwise deletion approach (complete case analysis) in which 5/235 (2.1%) patients in the erythromycin group and 13/470 (2.8%) patients in the control group were excluded from the analyses (flowchart depicted in Supplementary Fig. 1). Baseline characteristics assumed missing at random had >5% missing (PaO2/FiO2 ratio) were only used for descriptive purposes.

The proportions of patients lost to follow-up for the primary outcome of mortality were low and equally distributed among treatment and control groups (7/235 [3.0%] in the treatment group and 16/470 [3.4%] in the control group at day 90; Supplementary Table 3). These patients were censored on the day last known to be alive in survival analyses. In the secondary clinical outcomes, duration of mechanical ventilation was not reported in patients not mechanically ventilated at admission. The proportion of missing values in the change in SOFA from baseline to day 4 score outcomes (ΔSOFA) was fairly high (123/705 [17.4%]), mostly due to people leaving the ICU. This may slightly bias the results for the ΔSOFA, but is unlikely to change the conclusion (no difference between groups).

***Host response biomarker assays and handling of missing biomarker data***

Ethylenediaminetetraacetic acid-anticoagulated plasma (left-over after routine clinical laboratory measurements) was obtained from patients at admission (within 16 hours of admission), day 2 and day 4. Supplementary Table 2 provides an overview of the biomarkers used in this study. Biomarkers were measured using either FlexSet cytometric bead array (BD Biosciences, San Jose, California) on a FACS Calibur (Becton Dickenson, Frankin Lakes, New Jersey), or Luminex multiplex assays (from R&D Systems, Abingdon, United Kigdom, or Procartaplex, eBioscience, San Diego, California) on a BioPlex 200 (BioRad, Hercules, California). These biomarkers were supplemented with selected measurements from routine clinical laboratory measurements: platelet counts, and prothrombin time (PT).

Supplementary Table 4 displays the number and proportions of missing biomarker data in the PS matched biomarker cohort per group per timepoint (admission or day 4). None of the individual biomarkers exceeded 10% missingness. We assumed these values to be missing at random, and therefore used linear mixed models (as described in the main manuscript) to account for missingness.

## Analysis of secondary clinical outcomes

For the secondary clinical outcomes, we assessed differences between groups using statistical tests that take into account the matched or weighted nature of the data. After PS matching, we used paired *t-*tests for normally distributed continuous variables, Wilcoxon’s signed-rank for non-normally distributed continuous variables, and McNemar’s test for categorical data. After PS weighting, we used *t*-tests for normally distributed continuous variables, Wilcoxon’s rank-sum for non-normally distributed continuous variables, and the chi-squared test for categorical data (as provided in the *survey* R package) [10].

## Analysis of host response biomarkers

Patients in whom host response biomarkers had been measured were PS matched. To calculate the PS in this population, we used the same covariates as in the model used for mortality, because host response aberrations in inflammation, endothelial cell activation, and coagulation are affected by many of the same (potential) confounders, such as disease severity, site of infection, and comorbidities [11,12].

We used linear mixed models with log2-transformed biomarker levels as the dependent variables to examine whether erythromycin affects host response biomarkers over time between admission (when no patients had received erythromycin) and day 4 (when all patients in the treatment group had received erythromycin). The independent variables were treatment, time point (admission or day 4), and the interaction between treatment and time point. The model included a random intercept and slope, allowing the change over time to vary per individual patient. This also allowed us to include patients with missing biomarker values at either admission (at least one missing biomarker in 19/150 [12.7%] treated patients and 17/150 [11.3%] control patients) or day 4 (at least one missing biomarker in 22/143 [15.4%] of treated patients and 16/139 [11.5%] of control patients; considered missing at random, see Supplementary Methods and Supplementary Table 4). We were primarily interested in the P-values for the differences between the groups at day 4 – obtained as the P-value for the treatment coefficient in models using day 4, rather than admission, as the reference category – and the interaction term for treatment group and time.

## Calculation of E-Values to assess residual confounding

Residual (unmeasured) confounding may play a role in non-randomized studies of interventions, even when carefully addressing common forms of selection bias and controlling for (measured) confounding variables based on a causal diagram/directed acyclic graph. The E-Value is a novel method to assess the potential impact of residual confounding. It “represents the minimum strength of association, on the risk ratio scale, that an unmeasured confounder would need to have with both the treatment and outcome to fully explain away a specific treatment–outcome association, conditional on the measured covariates” [13]. Because our treatment-outcome association did not reject the null-hypothesis (no difference between treated and untreated patients), we calculated E-Values that represent the strength of association needed for an unmeasured confounder to either move the lower limit of the 95% CI to >1.00 (which would indicate a harmful effect of treatment) or move the upper limit of the 95% CI to <1.00 (which would indicate a beneficial effect of treatment). We did this using the equation for calculating E-Values based on hazard ratios for common outcomes (occurring in >15% of patients), as provided in the “EValue” R package [14].

The E-Values for mortality up to day 90 in the PS matched and weighted populations of our primary analysis range from 1.61 to 2.08 (for a beneficial or harmful effect of treatment on 90-day mortality in the PS matched population, respectively; Supplementary Table 6). The interpretation of the E-Value depends on the context of the research and analysis in which it is being applied [13]. In our study, it seems unlikely that after controlling for a large number of (potential) confounders, any unmeasured confounders remain that could result in consistent rejection of the null-hypothesis.

## Competing risk analysis

As the hazard for death at any point during follow-up is likely to be higher in patients admitted to the ICU than patients not in the ICU, we performed competing risk analysis to assess whether later ICU discharge observed in the erythromycin group in the unadjusted analysis affected the estimates for the effect of erythromycin treatment on mortality. We calculated cause-specific hazard ratios by using Cox proportional hazard models censoring patients in whom an event other than the event of interest occurred first. We calculated subdistribution hazard ratios as specified by Fine and Gray [15]. To account for the matched pairs in the PS matched population, we calculated the standard error of the cause-specific hazard ratio with a robust variance estimator [16], and used a clustered Fine-Gray model to calculate the subdistribution hazard ratios [17].

## R packages

The R packages used for statistical analyses included: MatchIt, WeightIt, cobalt, survey, RISCA, survival, survminer, nlme, EValue, rstatix, broom, cmprsk, crrSC, and lmtest.

**SUPPLEMENTARY TABLES**

***Supplementary table 1. Study protocol for the target trial and its emulation***

|  | **Target trial specification** | **Target trial emulation** |
| --- | --- | --- |
| **Eligibility criteria** | - Sepsis (as defined by sepsis-3 criteria) - No readmissions - No transfers from other hospitals - Not using azithromycin, clarithromycin, or high-dose erythromycin (≥500mg) at eligibility screening - No contra-indications for erythromycin use | Same, but patients had to be alive and in the ICU during the exposure period of 72 hours. |
| **Treatment strategies** | 1. Low-dose erythromycin (125-250mg/administration) 2. No low-dose erythromycin | Same strategies, but erythromycin prescribed for gastrointestinal dysmotility |
| **Assignment procedures** | Random assignment | Assigned to erythromycin group if started within a exposure period of 72 hours. Confounders balanced using PS methods |
| **Follow-up period** | Starts at assignment to treatment group, up to 90 days after ICU admission | Starts after 72 hour exposure period, up to 90 days after ICU admission |
| **Primary outcome** | Mortality up to 90 days | Same. |
| **Causal contrast of interest** | Per protocol analysis  Intention-to-treat analysis | Same* |
| **Analysis plan** | Estimate absolute and relative differences in mortality | Same, but estimate ATT and ATE separately |
| * exact per protocol treatment strategy is put in perspective in discussion section.  ATE = average treatment effect; ATT = average treatment effect on the treated; PS = propensity score | | |

***Supplementary Table 2. Overview of host response biomarker measurements***

|  | **Assay / platform** |
| --- | --- |
| **INFLAMMATION** |  |
| Interleukin-6 | FlexSet cytometric bead array* on FACS Calibur^†^ |
| Interleukin-8 | FlexSet cytometric bead array* on FACS Calibur^†^ |
| Interleukin-10 | FlexSet cytometric bead array* on FACS Calibur^†^ |
| Matrix metalloproteinase 8 | Luminex^‡^ on BioPlex 200^§^ |
| **ENDOTHELIAL CELL ACTIVATION** | |
| E-Selectin | FlexSet cytometric bead array* on FACS Calibur^†^ |
| Intercellular adhesion molecule 1 | FlexSet cytometric bead array* on FACS Calibur^†^ |
| Fractalkine | FlexSet cytometric bead array* on FACS Calibur^†^ |
| Angiopoetin-1 | Luminex^‡^ on BioPlex 200^§^ |
| Angiopoetin-2 | Luminex^‡^ on BioPlex 200^§^ |
| **COAGULATION** |  |
| D-dimer | Luminex^‡^ on BioPlex 200^§^ |
| Prothrombin time | Photometric method with Dade Innovin Reagent^‖^ |
| Protein C | Luminex^‡^ on BioPlex 200^§^ |
| Antithrombin | Luminex^‡^ on BioPlex 200^§^ |
| Platelets | Hemocytometry |
| *BD Biosciences, San Jose, California  ^†^ Becton Dickenson, Frankin Lakes, New Jersey  ^‡^R&D Systems, Abingdon, United Kigdom, or Procartaplex, eBioscience, San Diego, California  ^§^BioRad, Hercules, California  ^‖^Siemens Healthcare Diagnostics | |

***Supplementary Table 3. Overview of missing clinical data***

|  | **UNADJUSTED** | | **PS MATCHED** | | **PS WEIGHTED** | |  |
| --- | --- | --- | --- | --- | --- | --- | --- |
|  | **Erythromycin  (n = 235)** | **Controls  (n = 470)** | **Erythromycin  (n = 211)** | **Controls  (n = 211)** | **Erythromycin** | **Controls** | **Missingness classification*** |
| **DEMOGRAPHICS** |  |  |  |  |  |  |  |
| Age | 0 | 0 | 0 | 0 | 0 | 0 |  |
| Sex | 0 | 0 | 0 | 0 | 0 | 0 |  |
| Body mass index | 1 (0.4) | 7 (1.5) | 0 | 0 | 0 | 0 | MCAR |
| Race | 1 (0.4) | 1 (0.2) | 1 (0.5) | 0 | 0.5 | 0.2 | MCAR |
| **ADMISSION DATA** |  |  |  |  |  |  |  |
| Hospital A | 0 | 0 | 0 | 0 | 0 | 0 |  |
| Admission type, surgical | 0 | 0 | 0 | 0 | 0 | 0 |  |
| SDD use during admission | 0 | 0 | 0 | 0 | 0 | 0 |  |
| **CHRONIC COMORBIDITIES** |  |  |  |  |  |  |  |
| Charlson Comorbidity Index (no age) | 0 | 0 | 0 | 0 | 0 | 0 |  |
| Any malignancy | 0 | 0 | 0 | 0 | 0 | 0 |  |
| Non-metastatic solid tumor | 0 | 0 | 0 | 0 | 0 | 0 |  |
| Metastatic malignancy | 0 | 0 | 0 | 0 | 0 | 0 |  |
| Hematologic malignancy | 0 | 0 | 0 | 0 | 0 | 0 |  |
| Diabetes mellitus (type 1 or type 2) | 0 | 0 | 0 | 0 | 0 | 0 |  |
| Cerebrovascular disease | 0 | 0 | 0 | 0 | 0 | 0 |  |
| Hemiplegia | 0 | 0 | 0 | 0 | 0 | 0 |  |
| **CHRONIC MEDICATION** |  |  |  |  |  |  |  |
| Any immunosuppressant | 1 (0.4) | 15 (3.2) | 1 (0.5) | 6 (2.8) | 0.7 | 2.6 | MCAR |
| Antiplatelet drugs | 1 (0.4) | 15 (3.2) | 1 (0.5) | 6 (2.8) | 0.7 | 2.6 | MCAR |
| Calcium-entry blockers | 0 | 1 (0.2) | 0 | 1 (0.5) | 0 | 0.2 | MCAR |
| Beta-adrenergic blockers | 0 | 1 (0.2) | 0 | 1 (0.5) | 0 | 0.2 | MCAR |
| Oral antidiabetic drugs | 0 | 1 (0.2) | 0 | 1 (0.5) | 0 | 0.2 | MCAR |
| Insulin | 0 | 1 (0.2) | 0 | 1 (0.5) | 0 | 0.2 | MCAR |
| **DISEASE SEVERITY AT ICU ADMISSION** |  |  |  |  |  |  |  |
| APACHE IV score | 0 | 0 | 0 | 0 | 0 | 0 |  |
| Acute physiology score | 0 | 0 | 0 | 0 | 0 | 0 |  |
| mSOFA score | 4 (1.7) | 5 (1.1) | 0 | 0 | 0 | 0 |  |
| Shock | 2 (0.9) | 1 (0.2) | 0 | 0 | 0 | 0 |  |
| ARDS | 0 | 0 | 0 | 0 | 0 | 0 |  |
| AKI | 0 | 0 | 0 | 0 | 0 | 0 |  |
| Gastrointestinal Failure score | 0 | 1 (0.2) | 0 | 0 | 0 | 0 |  |
| Gastrointestinal bleeding | 0 | 0 | 0 | 0 | 0 | 0 |  |
| Mechanically ventilated | 2 (0.9) | 1 (0.2) | 0 | 0 | 0 | 0 |  |
| PaO2/FiO2 ratio | 10 (4.3) | 50 (10.6) | 10 (4.7) | 10 (4.7) | 6.8 | 8.2 | MAR |
| **SOURCE OF INFECTION** |  |  |  |  |  |  |  |
| Pulmonary tract | 0 | 0 | 0 | 0 | 0 | 0 |  |
| Abdominal tract | 0 | 0 | 0 | 0 | 0 | 0 |  |
| Urinary tract | 0 | 0 | 0 | 0 | 0 | 0 |  |
| Cardiovascular | 0 | 0 | 0 | 0 | 0 | 0 |  |
| Skin | 0 | 0 | 0 | 0 | 0 | 0 |  |
| Central nervous system | 0 | 0 | 0 | 0 | 0 | 0 |  |
| Other or unknown | 0 | 0 | 0 | 0 | 0 | 0 |  |
| **PRIMARY CLINICAL OUTCOME** |  |  |  |  |  |  |  |
| 30-day mortality | 3 (1.3) | 9 (1.9) | 3 (1.4) | 6 (2.8) | 1.0 | 2.1 | MCAR |
| 90-day mortality | 7 (3.0) | 16 (3.4) | 7 (3.3) | 9 (4.3) | 2.1 | 3.5 | MCAR |
| **SECONDARY CLINICAL OUTCOMES** |  |  |  |  |  |  |  |
| ICU length of stay | 0 | 0 | 0 | 0 | 0 | 0 |  |
| Hospital length of stay | 0 | 2 (0.4) | 0 | 1 (0.5) | 0 | 0.4 | MCAR |
| Duration of mechanical ventilation | 10 (4.3) | 51 (10.9) | 8 (3.8) | 8 (3.8) | 5.9 | 8.1 | MNAR† |
| ΔSOFA day 4 | 21 (8.9) | 102 (21.7) | 16 (7.6) | 32 (15.2) | 7.5 | 18.9 | MAR |
| Incidence of ICU-acquired infections | 0 | 0 | 0 | 0 | 0 | 0 |  |
| Incidence of ICU-acquired AKI | 0 | 0 | 0 | 0 | 0 | 0 |  |
| Incidence of ICU-acquired ARDS | 0 | 0 | 0 | 0 | 0 | 0 |  |
| MAR = missing at random; MCAR = missing completely at random; MNAR = missing not at random.  Variables are displayed as count (percentage) for unadjusted and PS matched data, and percentage for PS weighted data.  *All variables with ≤5% overall missing values were considered MCAR  †Duration of mechanical ventilation is only missing in patients not mechanically ventilated at ICU admission | | | | | | | |

***Supplementary Table 4. Overview of missing biomarker data***

|  | **Admission** | | **Day 4** | |  |
| --- | --- | --- | --- | --- | --- |
|  | **Erythromycin  (n = 150)** | **Controls  (n = 150)** | **Erythromycin  (n = 143)** | **Controls  (n = 139)** | **Missingness classification*** |
| **INFLAMMATION** | | | | | |
| IL-6 | 14 (9.3) | 14 (9.3) | 14 (9.8) | 9 (6.5) | MAR |
| IL-8 | 14 (9.3) | 14 (9.3) | 14 (9.8) | 9 (6.5) | MAR |
| IL-10 | 14 (9.3) | 14 (9.3) | 14 (9.8) | 9 (6.5) | MAR |
| MMP-8 | 14 (9.3) | 14 (9.3) | 14 (9.8) | 10 (7.2) | MAR |
| **ENDOTHELIAL CELL ACTIVATION** | | | | | |
| E-Selectin | 14 (9.3) | 14 (9.3) | 14 (9.8) | 9 (6.5) | MAR |
| ICAM-1 | 14 (9.3) | 14 (9.3) | 14 (9.8) | 9 (6.5) | MAR |
| Fractalkine | 14 (9.3) | 14 (9.3) | 14 (9.8) | 9 (6.5) | MAR |
| Angiopoetin-1 | 14 (9.3) | 14 (9.3) | 14 (9.8) | 10 (7.2) | MAR |
| Angiopoetin-2 | 14 (9.3) | 14 (9.3) | 14 (9.8) | 10 (7.2) | MAR |
| Angiopoetin-1/ Angiopoetin-2 ratio | 14 (9.3) | 14 (9.3) | 14 (9.8) | 10 (7.2) | MAR |
| **COAGULATION** | | | | | |
| D-dimer | 14 (9.3) | 14 (9.3) | 14 (9.8) | 10 (7.2) | MAR |
| Prothrombin time | 6 (4.0) | 5 (3.3) | 12 (8.4) | 7 (5.0) | MAR |
| Protein C | 14 (9.3) | 14 (9.3) | 14 (9.8) | 10 (7.2) | MAR |
| Antithrombin | 14 (9.3) | 14 (9.3) | 14 (9.8) | 10 (7.2) | MAR |
| Platelets | 3 (2.0) | 2 (1.3) | 4 (2.8) | 2 (1.4) | MCAR |
| MAR = missing at random; MCAR = missing completely at random.  Variables are displayed as count (percentage)  *All variables with ≤5% overall missing values were considered MCAR | | | | | |

***Supplementary Table 5. PS matched and weighted populations for the primary analysis***

|  | **PS MATCHED** | | | **PS WEIGHTED** | | |
| --- | --- | --- | --- | --- | --- | --- |
|  | **Erythromycin**  **(n = 211)** | **Controls**  **(n = 211)** | **SMD*** | **Erythromycin** | **Controls** | **SMD*** |
| **DEMOGRAPHICS** |  |  |  |  |  |  |
| Age, years | 61.3 (13.9) | 60.5 (14.5) | 0.061 | 61.1 (13.8) | 60.8 (15.0) | 0.019 |
| Sex, male | 142 (67.3) | 145 (68.7) | 0.030 | 67.4 | 63.4 | 0.083 |
| Body mass index | 25.3 [23.0, 29.3] | 26.0 [23.1, 29.5] | 0.023 | 24.9 [22.6, 28.7] | 25.7 [22.9, 29.2] | 0.008 |
| Race, white | 184 (87.6) | 196 (92.9) | 0.178 | 88.5 | 90.3 | 0.057 |
| **ADMISSION DATA** |  |  |  |  |  |  |
| Hospital A | 129 (61.1) | 124 (58.8) | 0.048 | 56.2 | 54.5 | 0.034 |
| Admission type, surgical | 66 (31.3) | 64 (30.3) | 0.021 | 29.0 | 27.4 | 0.035 |
| SDD use during admission | 163 (77.3) | 144 (68.2) | 0.203 | 76.1 | 69.5 | 0.148 |
| **CHRONIC COMORBIDITIES** |  |  |  |  |  |  |
| Charlson Comorbidity Index (no age) | 2 [0, 4] | 2 [0, 3] | 0.013 | 2 [0, 3] | 2 [0, 3] | 0.005 |
| Any malignancy | 50 (23.7) | 52 (24.6) | 0.022 | 21.6 | 22.7 | 0.027 |
| Non-metastatic solid tumor | 30 (14.2) | 24 (11.4) | 0.085 | 12.7 | 10.9 | 0.055 |
| Metastatic malignancy | 5 (2.4) | 10 (4.7) | 0.128 | 2.0 | 4.1 | 0.127 |
| Hematologic malignancy | 17 (8.1) | 19 (9.0) | 0.034 | 8.1 | 8.0 | 0.001 |
| Diabetes mellitus (type 1 or type 2) | 45 (21.3) | 46 (21.8) | 0.012 | 21.3 | 22.1 | 0.020 |
| Cerebrovascular disease | 24 (11.4) | 20 (9.5) | 0.062 | 11.9 | 9.3 | 0.083 |
| Hemiplegia | 5 (2.4) | 3 (1.4) | 0.070 | 3.8 | 3.1 | 0.034 |
| **CHRONIC MEDICATION** | |  |  |  |  |  |
| Any immunosuppressant | 28 (13.3) | 36 (17.6) | 0.117 | 14.0 | 16.0 | 0.057 |
| Antiplatelet drugs | 58 (27.6) | 61 (29.8) | 0.047 | 25.0 | 26.4 | 0.033 |
| Calcium-entry blockers | 38 (18.0) | 34 (16.2) | 0.048 | 17.8 | 16.9 | 0.024 |
| Beta-adrenergic blockers | 59 (28.0) | 62 (29.5) | 0.035 | 26.8 | 27.2 | 0.009 |
| Oral antidiabetic drugs | 27 (12.8) | 23 (11.0) | 0.057 | 12.8 | 13.5 | 0.021 |
| Insulin | 25 (11.8) | 28 (13.3) | 0.045 | 12.8 | 12.2 | 0.018 |
| **DISEASE SEVERITY AT ICU ADMISSION** | |  |  |  |  |  |
| APACHE IV score | 90.6 (29.2) | 91.5 (28.5) | 0.032 | 88.2 (28.4) | 86.8 (28.8) | 0.048 |
| Acute physiology score | 77.7 (27.0) | 78.3 (27.2) | 0.021 | 75.6 (26.4) | 73.8 (26.8) | 0.067 |
| mSOFA score | 8 [6, 11] | 8.0 [7, 10] | 0.016 | 8 [5, 10] | 8 [5, 9] | 0.050 |
| Shock | 157 (74.4) | 157 (74.4) | <0.001 | 62.7 | 60.2 | 0.051 |
| ARDS | 71 (33.6) | 69 (32.7) | 0.020 | 28.9 | 29.4 | 0.010 |
| AKI | 104 (49.3) | 104 (49.3) | <0.001 | 44.0 | 43.0 | 0.020 |
| Gastrointestinal Failure score |  |  | 0.167 | 50.9 |  | 0.131 |
| 0 - Normal gastrointestinal function | 83 (39.3) | 84 (39.8) |  | 20.7 | 49.2 |  |
| 1 - Reduced/delayed enteral feeding† | 95 (45.0) | 82 (38.9) |  | 6.4 | 33.5 |  |
| 2 - Food intolerance or IAH | 31 (14.7) | 43 (20.4) |  | 4.3 | 16.7 |  |
| 3 - Food intolerance and IAH | 2 (0.9) | 2 (0.9) |  | 4.8 | 0.6 |  |
| 4 - Abdominal compartment syndrome | 0 (0.0) | 0 (0.0) |  | 3.0 | 0.0 |  |
| Gastrointestinal Failure score >= 1 | 128 (60.7) | 127 (60.2) | 0.010 | 53.6 | 50.8 | 0.055 |
| Gastrointestinal bleeding | 6 (2.8) | 3 (1.4) | 0.099 | 3.3 | 1.4 | 0.121 |
| Mechanically ventilated | 203 (96.2) | 203 (96.2) | <0.001 | 94.1 | 91.9 | 0.087 |
| PaO2/FiO2 ratio | 148 [97, 230] | 148 [100, 207] | 0.055 | 156 [99, 237] | 152 [102, 213] | 0.073 |
| **SOURCE OF INFECTION** |  |  |  |  |  |  |
| Pulmonary tract | 109 (51.7) | 107 (50.7) | 0.019 | 50.9 | 52.5 | 0.032 |
| Abdominal tract | 45 (21.3) | 41 (19.4) | 0.047 | 20.7 | 18.9 | 0.046 |
| Urinary tract | 18 (8.5) | 16 (7.6) | 0.035 | 6.4 | 6.4 | <0.001 |
| Cardiovascular | 10 (4.7) | 11 (5.2) | 0.022 | 4.3 | 4.3 | 0.002 |
| Skin | 14 (6.6) | 13 (6.2) | 0.019 | 4.8 | 5.0 | 0.007 |
| Central nervous system | 4 (1.9) | 5 (2.4) | 0.033 | 3.0 | 4.6 | 0.087 |
| Other or unknown | 26 (12.3) | 31 (14.7) | 0.069 | 15.9 | 14.3 | 0.046 |
| AKI = acute kidney injury; APACHE-IV = acute physiology and chronic health evaluation IV; ARDS = acute respiratory distress syndrome; IAH = intraabdominal hypertension; ICU = intensive care unit; mSOFA = modified sequential organ failure assessment score (without the central nervous system component); SMD = standardized mean difference.  *SMD >0.2 indicates a substantial imbalance between groups; <0.1 indicates a negligible difference  † “Enteral feeding <50% of calculated needs or no feeding 3 days after abdominal surgery” in the original paper.[18] | | | | | | |

## Supplementary Table 6. E-Values to shift hazard ratios of the primary analysis to a range consistent with harm or benefit

|  | **90-day mortality rate** | |
| --- | --- | --- |
|  | **Harm** | **Benefit** |
| **PS MATCHED** | 2.08 | 1.61 |
| **PS WEIGHTED** | 2.02 | 1.80 |
| These E-Values represent the strength of association (on the relative risk scale)* needed for an unmeasured confounder to either move the lower limit of the 95% CI to >1.00 (harm) or move the upper limit of the 95% CI to <1.00 (benefit).  *calculated based on hazard ratios for common outcomes (occurring in >15% of patients), with equations provided in the “EValue” R package [14]. | | |

***Supplementary Table 7. Baseline characteristics and PS matched population for the host response biomarker analyses***

|  | **BIOMARKER COHORT** | | | **PS MATCHED** | | |
| --- | --- | --- | --- | --- | --- | --- |
|  | **Erythromycin**  **(n = 172)** | **Controls**  **(n = 304)** | **SMD*** | **Erythromycin**  **(n = 150)** | **Controls**  **(n = 150)** | **SMD*** |
| **DEMOGRAPHICS** |  |  |  |  |  |  |
| Age, years | 61.4 (13.7) | 60.3 (15.3) | 0.075 | 61.3 (14.0) | 62.1 (13.9) | 0.059 |
| Sex, male | 119 (69.2) | 180 (59.2) | 0.209 | 101 (67.3) | 98 (65.3) | 0.042 |
| Body mass index | 25.9 [23.1, 29.4] | 25.4 [22.9, 29.0] | 0.095 | 25.8 [22.9, 29.4] | 25.8 [22.9, 29.2] | 0.019 |
| Race, white | 152 (88.9) | 265 (87.2) | 0.053 | 131 (87.9) | 138 (92.0) | 0.136 |
| **ADMISSION DATA** |  |  |  |  |  |  |
| Hospital A | 98 (57.0) | 128 (42.1) | 0.301 | 82 (54.7) | 76 (50.7) | 0.080 |
| Admission type, surgical | 48 (27.9) | 74 (24.3) | 0.081 | 43 (28.7) | 38 (25.3) | 0.075 |
| SDD use during admission | 130 (75.6) | 194 (63.8) | 0.258 | 112 (74.7) | 94 (62.7) | 0.261 |
| **CHRONIC COMORBIDITIES** |  |  |  |  |  |  |
| Charlson Comorbidity Index (no age) | 2 [0, 3] | 2 [0, 3] | 0.063 | 2 [0, 3] | 2 [0, 3] | 0.003 |
| Any malignancy | 41 (23.8) | 67 (22.0) | 0.043 | 37 (24.7) | 37 (24.7) | <0.001 |
| Non-metastatic solid tumor | 19 (11.0) | 26 (8.6) | 0.084 | 17 (11.3) | 14 (9.3) | 0.066 |
| Metastatic malignancy | 5 (2.9) | 12 (3.9) | 0.057 | 4 (2.7) | 6 (4.0) | 0.074 |
| Hematologic malignancy | 21 (12.2) | 31 (10.2) | 0.064 | 20 (13.3) | 17 (11.3) | 0.061 |
| Diabetes mellitus (type 1 or type 2) | 34 (19.8) | 65 (21.4) | 0.040 | 30 (20.0) | 39 (26.0) | 0.143 |
| Cerebrovascular disease | 21 (12.2) | 25 (8.2) | 0.132 | 19 (12.7) | 14 (9.3) | 0.107 |
| Hemiplegia | 4 (2.3) | 11 (3.6) | 0.076 | 3 (2.0) | 4 (2.7) | 0.044 |
| **CHRONIC MEDICATION** | |  |  |  |  |  |
| Any immunosuppressant | 24 (14.0) | 52 (17.9) | 0.105 | 19 (12.8) | 28 (19.4) | 0.183 |
| Antiplatelet drugs | 43 (25.1) | 73 (25.1) | 0.001 | 38 (25.5) | 37 (25.7) | 0.004 |
| Calcium-entry blockers | 33 (19.2) | 53 (17.5) | 0.044 | 26 (17.3) | 31 (20.8) | 0.088 |
| Beta-adrenergic blockers | 44 (25.6) | 81 (26.7) | 0.026 | 39 (26.0) | 40 (26.8) | 0.019 |
| Oral antidiabetic drugs | 24 (14.0) | 38 (12.5) | 0.042 | 23 (15.3) | 20 (13.4) | 0.054 |
| Insulin | 16 (9.3) | 35 (11.6) | 0.074 | 13 (8.7) | 19 (12.8) | 0.132 |
| **DISEASE SEVERITY AT ICU ADMISSION** | |  |  |  |  |  |
| APACHE IV score | 89.5 (27.2) | 87.2 (28.5) | 0.085 | 89.2 (27.6) | 89.7 (27.7) | 0.017 |
| Acute physiology score | 76.5 (25.2) | 74.1 (26.5) | 0.092 | 76.2 (25.6) | 75.9 (26.2) | 0.011 |
| mSOFA score | 8 [7, 11] | 7 [5, 9] | 0.394 | 8 [6, 10] | 8 [7, 10] | 0.004 |
| Shock | 132 (76.7) | 167 (54.9) | 0.473 | 111 (74.0) | 109 (72.7) | 0.030 |
| ARDS | 71 (41.3) | 110 (36.2) | 0.105 | 60 (40.0) | 65 (43.3) | 0.068 |
| AKI | 86 (50.0) | 138 (45.4) | 0.092 | 76 (50.7) | 78 (52.0) | 0.027 |
| Gastrointestinal Failure score |  |  | 0.369 |  |  | 0.157 |
| 0 - Normal gastrointestinal function | 64 (37.2) | 162 (53.3) |  | 58 (38.7) | 59 (39.3) |  |
| 1 - Reduced/delayed enteral feeding† | 77 (44.8) | 89 (29.3) |  | 65 (43.3) | 56 (37.3) |  |
| 2 - Food intolerance or IAH | 29 (16.9) | 50 (16.4) |  | 26 (17.3) | 33 (22.0) |  |
| 3 - Food intolerance and IAH | 2 (1.2) | 2 (0.7) |  | 1 (0.7) | 2 (1.3) |  |
| 4 - Abdominal compartment syndrome | 0 (0.0) | 0 (0.0) |  | 0 (0.0) | 0 (0.0) |  |
| Gastrointestinal Failure score >= 1 | 108 (62.8) | 141 (46.5) | 0.331 | 92 (61.3) | 91 (60.7) | 0.014 |
| Gastrointestinal bleeding | 3 (1.7) | 4 (1.3) | 0.035 | 3 (2.0) | 2 (1.3) | 0.052 |
| Mechanically ventilated | 165 (95.9) | 279 (91.8) | 0.174 | 143 (95.3) | 145 (96.7) | 0.068 |
| PaO2/FiO2 ratio | 136 [93, 206] | 153 [101, 216] | 0.096 | 136 [92, 208] | 144 [94, 194] | 0.060 |
| **SOURCE OF INFECTION** |  |  |  |  |  |  |
| Pulmonary tract | 90 (52.3) | 161 (53.0) | 0.013 | 78 (52.0) | 82 (54.7) | 0.053 |
| Abdominal tract | 39 (22.7) | 59 (19.4) | 0.080 | 37 (24.7) | 32 (21.3) | 0.079 |
| Urinary tract | 13 (7.6) | 15 (4.9) | 0.109 | 11 (7.3) | 13 (8.7) | 0.049 |
| Cardiovascular | 13 (7.6) | 15 (4.9) | 0.109 | 11 (7.3) | 10 (6.7) | 0.026 |
| Skin | 15 (8.7) | 11 (3.6) | 0.213 | 8 (5.3) | 9 (6.0) | 0.029 |
| Central nervous system | 3 (1.7) | 16 (5.3) | 0.192 | 3 (2.0) | 3 (2.0) | <0.001 |
| Other or unknown | 16 (9.3) | 52 (17.1) | 0.232 | 16 (10.7) | 13 (8.7) | 0.068 |
| AKI = acute kidney injury; APACHE-IV = acute physiology and chronic health evaluation IV; ARDS = acute respiratory distress syndrome; IAH = intraabdominal hypertension; ICU = intensive care unit; mSOFA = modified sequential organ failure assessment score (without the central nervous system component); SMD = standardized mean difference.  *SMD >0.2 indicates a substantial imbalance between groups; <0.1 indicates a negligible difference  † “Enteral feeding <50% of calculated needs or no feeding 3 days after abdominal surgery” in the original paper.[18] | | | | | | |

***Supplementary Table 8. Baseline characteristics for the intention-to-treat sensitivity analysis***

|  | **Erythromycin**  **(n = 235)** | **Controls**  **(n = 571)** | ***P-*value** | **SMD*** |
| --- | --- | --- | --- | --- |
| **DEMOGRAPHICS** |  |  |  |  |
| Age, years | 60.8 (13.6) | 60.3 (15.4) | 0.659 | 0.033 |
| Sex, male | 162 (68.9) | 344 (60.2) | 0.020 | 0.182 |
| Body mass index | 25.5 [23.0, 29.3] | 25.2 [22.6, 28.8] | 0.238 | 0.104 |
| Race, white | 208 (88.9) | 510 (89.5) | 0.803 | 0.019 |
| **ADMISSION DATA** |  |  |  |  |
| Hospital A | 149 (63.4) | 289 (50.6) | 0.001 | 0.261 |
| Admission type, surgical | 75 (31.9) | 140 (24.5) | 0.035 | 0.165 |
| SDD use during admission | 181 (77.0) | 396 (69.4) | 0.032 | 0.174 |
| **CHRONIC COMORBIDITIES** |  |  |  |  |
| Charlson Comorbidity Index (no age) | 2 [0, 4] | 2.0 [0, 3] | 0.269 | 0.075 |
| Any malignancy | 58 (24.7) | 126 (22.1) | 0.460 | 0.062 |
| Non-metastatic solid tumor | 33 (14.0) | 62 (10.9) | 0.229 | 0.097 |
| Metastatic malignancy | 6 (2.6) | 21 (3.7) | 0.521 | 0.065 |
| Hematologic malignancy | 23 (9.8) | 46 (8.1) | 0.410 | 0.061 |
| Diabetes mellitus (type 1 or type 2) | 49 (20.9) | 110 (19.3) | 0.627 | 0.040 |
| Cerebrovascular disease | 28 (11.9) | 55 (9.6) | 0.372 | 0.074 |
| Hemiplegia | 6 (2.6) | 18 (3.2) | 0.821 | 0.036 |
| **CHRONIC MEDICATION** |  |  |  |  |
| Any immunosuppressant | 32 (13.7) | 87 (15.7) | 0.514 | 0.057 |
| Antiplatelet drugs | 63 (26.9) | 143 (25.8) | 0.790 | 0.025 |
| Calcium-entry blockers | 43 (18.3) | 94 (16.5) | 0.537 | 0.048 |
| Beta-adrenergic blockers | 65 (27.7) | 160 (28.1) | 0.931 | 0.009 |
| Oral antidiabetic drugs | 29 (12.3) | 68 (11.9) | 0.905 | 0.013 |
| Insulin | 26 (11.1) | 59 (10.4) | 0.801 | 0.023 |
| **DISEASE SEVERITY AT ICU ADMISSION** |  |  |  |  |
| APACHE IV score | 90.9 (28.5) | 86.2 (28.1) | 0.031 | 0.168 |
| Acute physiology score | 78.2 (26.4) | 73.3 (26.2) | 0.017 | 0.186 |
| mSOFA score | 8 [6, 11] | 7 [5, 9] | <0.001 | 0.347 |
| Shock | 176 (75.5) | 309 (54.5) | <0.001 | 0.452 |
| ARDS | 78 (33.2) | 175 (30.6) | 0.504 | 0.055 |
| AKI | 117 (49.8) | 233 (40.8) | 0.023 | 0.181 |
| Gastrointestinal Failure score |  |  | <0.001 | 0.431 |
| 0 - Normal gastrointestinal function | 86 (36.6) | 313 (54.8) |  |  |
| 1 - Reduced/delayed enteral feeding† | 112 (47.7) | 166 (29.1) |  |  |
| 2 - Food intolerance or IAH | 35 (14.9) | 87 (15.2) |  |  |
| 3 - Food intolerance and IAH | 2 (0.9) | 2 (0.4) |  |  |
| 4 - Abdominal compartment syndrome | 0 (0.0) | 0 (0.00 |  |  |
| Gastrointestinal Failure score >= 1 | 149 (63.4) | 255 (44.9) | <0.001 | 0.378 |
| Gastrointestinal bleeding | 7 (3.0) | 12 (2.1) | 0.451 | 0.056 |
| Mechanically ventilated | 225 (96.6) | 513 (90.5) | 0.003 | 0.249 |
| PaO2/FiO2 ratio | 148 [99, 230] | 152 [104, 214] | 0.873 | 0.025 |
| **SOURCE OF INFECTION** |  |  |  |  |
| Pulmonary tract | 119 (50.6) | 302 (52.9) | 0.587 | 0.045 |
| Abdominal tract | 48 (20.4) | 112 (19.6) | 0.846 | 0.020 |
| Urinary tract | 20 (8.5) | 32 (5.6) | 0.155 | 0.114 |
| Cardiovascular | 15 (6.4) | 18 (3.2) | 0.049 | 0.152 |
| Skin | 17 (7.2) | 20 (3.5) | 0.026 | 0.166 |
| Central nervous system | 6 (2.6) | 35 (6.1) | 0.035 | 0.176 |
| Other or unknown | 30 (12.8) | 91 (15.9) | 0.279 | 0.091 |
| AKI = acute kidney injury; APACHE-IV = acute physiology and chronic health evaluation IV; ARDS = acute respiratory distress syndrome; IAH = intraabdominal hypertension; ICU = intensive care unit; mSOFA = modified sequential organ failure assessment score (without the central nervous system component); SMD = standardized mean difference.  *SMD >0.2 indicates a substantial imbalance between groups; <0.1 indicates a negligible difference  † “Enteral feeding <50% of calculated needs or no feeding 3 days after abdominal surgery” in the original paper.[18]  Categorical data are displayed as count (percentage) and compared using Fisher's exact test.  Normally distributed continuous data are displayed as mean (standard deviation) and compared using Welch's *t*-test.  Non-normally distributed continuous data are displayed as median [interquartile range] and compared using Wilcoxon's rank-sum test. | | | | |
|  |  |  |  |  |

***Supplementary Table 9. PS matched and PS weighted populations for the intention-to-treat sensitivity analysis***

|  | **PS MATCHED** | | | **PS WEIGHTED** | | |
| --- | --- | --- | --- | --- | --- | --- |
|  | **Erythromycin**  **(n = 224)** | **Controls**  **(n = 224)** | **SMD*** | **Erythromycin** | **Controls** | **SMD*** |
| **DEMOGRAPHICS** |  |  |  |  |  |  |
| Age, years | 61.0 (13.7) | 59.7 (14.4) | 0.093 | 60.6 (13.9) | 60.4 (15.2) | 0.014 |
| Sex, male | 152 (67.9) | 154 (68.8) | 0.019 | 66.2 | 62.7 | 0.073 |
| Body mass index | 25.3 [23.0, 29.3] | 25.3 [22.5, 29.1] | 0.029 | 24.9 [22.5, 28.7] | 25.4 [22.8, 29.0] | 0.011 |
| Race, white | 197 (88.3) | 210 (93.8) | 0.190 | 88.1 | 90.7 | 0.085 |
| **ADMISSION DATA** |  |  |  |  |  |  |
| Hospital A | 141 (62.9) | 141 (62.9) | <0.001 | 56.4 | 54.5 | 0.039 |
| Admission type, surgical | 69 (30.8) | 63 (28.1) | 0.059 | 28.8 | 26.9 | 0.043 |
| SDD use during admission | 171 (76.3) | 163 (72.8) | 0.093 | 76.1 | 69.5 | 0.148 |
| **CHRONIC COMORBIDITIES** |  |  |  |  |  |  |
| Charlson Comorbidity Index (no age) | 2 [0, 3] | 2 [0, 3] | 0.006 | 2 [0, 3] | 2 [0, 3] | 0.002 |
| Any malignancy | 56 (25.0) | 58 (25.9) | 0.021 | 21.9 | 22.8 | 0.022 |
| Non-metastatic solid tumor | 32 (14.3) | 27 (12.1) | 0.066 | 12.4 | 11.5 | 0.028 |
| Metastatic malignancy | 6 (2.7) | 6 (2.7) | <0.001 | 2.0 | 3.8 | 0.105 |
| Hematologic malignancy | 22 (9.8) | 25 (11.2) | 0.044 | 8.7 | 8.1 | 0.021 |
| Diabetes mellitus (type 1 or type 2) | 45 (20.1) | 43 (19.2) | 0.022 | 20.3 | 20.1 | 0.006 |
| Cerebrovascular disease | 25 (11.2) | 23 (10.3) | 0.029 | 11.7 | 9.3 | 0.081 |
| Hemiplegia | 5 (2.2) | 5 (2.2) | <0.001 | 3.6 | 2.9 | 0.044 |
| **CHRONIC MEDICATION** | |  |  |  |  |  |
| Any immunosuppressant | 31 (13.9) | 39 (18.0) | 0.111 | 14.2 | 15.9 | 0.049 |
| Antiplatelet drugs | 60 (26.9) | 53 (24.4) | 0.057 | 24.9 | 26.1 | 0.029 |
| Calcium-entry blockers | 40 (17.9) | 36 (16.1) | 0.046 | 17.5 | 16.5 | 0.027 |
| Beta-adrenergic blockers | 61 (27.2) | 58 (26.0) | 0.028 | 26.7 | 28.4 | 0.037 |
| Oral antidiabetic drugs | 28 (12.5) | 23 (10.3) | 0.069 | 12.0 | 11.8 | 0.008 |
| Insulin | 24 (10.7) | 25 (11.2) | 0.016 | 12.2 | 11.1 | 0.032 |
| **DISEASE SEVERITY AT ICU ADMISSION** | |  |  |  |  |  |
| APACHE IV score | 90.9 (28.8) | 88.6 (27.4) | 0.082 | 88.8 (28.4) | 87.4 (28.1) | 0.048 |
| Acute physiology score | 78.0 (26.6) | 75.5 (25.3) | 0.097 | 76.2 (26.5) | 74.5 (26.2) | 0.067 |
| mSOFA score | 8 [6, 11] | 8 [6, 10] | 0.020 | 8 [5, 10] | 8 [5, 10] | 0.050 |
| Shock | 169 (75.4) | 167 (74.6) | 0.021 | 63.8 | 61.0 | 0.057 |
| ARDS | 77 (34.4) | 72 (32.1) | 0.047 | 30.7 | 31.3 | 0.013 |
| AKI | 113 (50.4) | 101 (45.1) | 0.107 | 43.4 | 42.8 | 0.011 |
| Gastrointestinal Failure score |  |  | 0.220 |  |  | 0.147 |
| 0 - Normal gastrointestinal function | 85 (37.9) | 79 (35.3) |  | 45.7 | 49.1 |  |
| 1 - Reduced/delayed enteral feeding† | 104 (46.4) | 91 (40.6) |  | 39.5 | 33.4 |  |
| 2 - Food intolerance or IAH | 33 (14.7) | 52 (23.2) |  | 13.9 | 17.1 |  |
| 3 - Food intolerance and IAH | 2 (0.9) | 2 (0.9) |  | 0.9 | 0.4 |  |
| 4 - Abdominal compartment syndrome | 0 (0.0) | 0 (0.0) |  | 0.0 | 0.0 |  |
| Gastrointestinal Failure score >= 1 | 139 (62.1) | 145 (64.7) | 0.056 | 54.3 | 50.9 | 0.067 |
| Gastrointestinal bleeding | 7 (3.1) | 4 (1.8) | 0.087 | 3.2 | 1.9 | 0.082 |
| Mechanically ventilated | 216 (96.4) | 214 (95.5) | 0.045 | 94.2 | 92.5 | 0.067 |
| PaO2/FiO2 ratio | 149 [98, 230] | 140 [100, 214] | 0.118 | 151 [97, 236] | 149 [102, 211] | 0.072 |
| **SOURCE OF INFECTION** |  |  |  |  |  |  |
| Pulmonary tract | 117 (52.2) | 118 (52.7) | 0.009 | 50.2 | 52.3 | 0.042 |
| Abdominal tract | 46 (20.5) | 50 (22.3) | 0.044 | 21.8 | 19.9 | 0.048 |
| Urinary tract | 19 (8.5) | 19 (8.5) | <0.001 | 6.3 | 6.5 | 0.008 |
| Cardiovascular | 14 (6.2) | 11 (4.9) | 0.058 | 4.0 | 3.9 | 0.005 |
| Skin | 15 (6.7) | 13 (5.8) | 0.037 | 4.8 | 4.8 | 0.002 |
| Central nervous system | 4 (1.8) | 5 (2.2) | 0.032 | 3.7 | 4.9 | 0.057 |
| Other or unknown | 28 (12.5) | 26 (11.6) | 0.027 | 16.3 | 14.5 | 0.047 |
| AKI = acute kidney injury; APACHE-IV = acute physiology and chronic health evaluation IV; ARDS = acute respiratory distress syndrome; IAH = intraabdominal hypertension; ICU = intensive care unit; mSOFA = modified sequential organ failure assessment score (without the central nervous system component); SMD = standardized mean difference.  *SMD >0.2 indicates a substantial imbalance between groups; <0.1 indicates a negligible difference  † “Enteral feeding <50% of calculated needs or no feeding 3 days after abdominal surgery” in the original paper.[18] | | | | | | |

***Supplementary Table 10. Hazard ratios for mortality up to day 90 for the intention-to-treat sensitivity analysis***

|  | **90-day mortality rate** | |
| --- | --- | --- |
| **UNADJUSTED** | **Events, n (%)** | **HR (95% CI)** |
| Erythromycin (n = 235) | 76 (33.3) | 0.86 (0.66, 1.12) |
| Controls (n = 571) | 213 (38.4) | 1.00 (ref) |
| **PS MATCHED** |  |  |
| Erythromycin (n = 224) | 73 (33.6) | 0.96 (0.69 - 1.34) |
| Controls (n = 224) | 77 (35.6) | 1.00 (ref) |
| **PS WEIGHTED** | **Events, %** |  |
| Erythromycin | 33.8 | 0.89 (0.62 - 1.27) |
| Controls | 38.6 | 1.00 (ref) |
| CI = confidence interval; HR = hazard ratio; IPTW = inverse probability of treatment weighting; PS = propensity score; ref = referent. | | |

***Supplementary Table 11. Secondary clinical outcomes for the intention-to-treat sensitivity analysis***

|  | **UNADJUSTED** | | | **PS MATCHED** | | | **PS WEIGHTED** | | |
| --- | --- | --- | --- | --- | --- | --- | --- | --- | --- |
|  | **Erythromycin  (n = 235)** | **Controls  (n = 571)** | ***P-*value** | **Erythromycin  (n = 224)** | **Controls  (n = 224)** | ***P-*value** | **Erythromycin** | **Controls** | ***P-*value** |
| **SECONDARY CLINICAL OUTCOMES** | | | |  | | |  | | |
| 30-day mortality | 60 (25.9) | 152 (27.1) | 0.791 | 59 (26.7) | 57 (26.0) | 0.919 | 27.1 | 27.5 | 0.903 |
| ICU length of stay, days | 8 [5, 14] | 8 [5, 13] | 0.200 | 8 [5, 14] | 8 [5, 15] | 0.949 | 8 [5, 14] | 8 [5, 14] | 0.929 |
| Duration of mechanical ventilation, days | 6 [4, 11] | 6 [3, 10] | 0.314 | 7 [4, 11] | 6 [4, 11] | 0.828 | 6 [4, 10] | 6 [3, 11] | 0.885 |
| ΔSOFA day 4 | -1.5 (2.9) | -1.2 (2.7) | 0.171 | -1.4 (2.9) | -1.6 (2.8) | 0.798 | -1.3 (2.8) | -1.2 (2.7) | 0.672 |
| Incidence of ICU-acquired infections | 42 (17.9) | 91 (15.9) | 0.531 | 40 (17.9) | 36 (16.1) | 0.804 | 13.9 | 16.6 | 0.354 |
| Incidence of ICU-acquired AKI | 17 (7.2) | 40 (7.0) | 0.881 | 17 (7.6) | 19 (8.5) | 0.868 | 6.7 | 7.3 | 0.764 |
| Incidence of ICU-acquired ARDS | 9 (3.8) | 23 (4.0) | >0.999 | 9 (4.0) | 11 (4.9) | 0.823 | 3.6 | 4.4 | 0.642 |
| AKI = acute kidney injury; ARDS = acute respiratory distress syndrome; ICU = intensive care unit; PS = propensity score; ΔSOFA = change in sequential organ failure assessment score from admission to day 2, 3, or 4.  Categorical data are displayed as count (percentage) or and compared using Fisher's exact test (unadjusted) or McNemar's test (after PS matching), or displayed as percentage and compared using a chi-square test† (after PS weighting).  Normally distributed continuous data are displayed as mean (standard deviation) and compared using a *t*-test (unadjusted), a paired *t*-test (after PS matching) or a *t*-test† (after PS weighting).  Non-normally distributed continuous data are displayed as median [interquartile range] and compared using Wilcoxon's rank-sum test (unadjusted), Wilcoxon's signed-rank test, or Wilcoxon rank-sum test† (after PS weighting).  †for weighted samples, as provided in the *survey* R package | | | | | | | | | |

***Supplementary Table 12. Baseline characteristics for the sensitivity analysis with an exposure period of 48 hours***

|  | **Erythromycin**  **(n = 191)** | **Controls**  **(n = 637)** | ***P-*value** | **SMD*** |
| --- | --- | --- | --- | --- |
| **DEMOGRAPHICS** |  |  |  |  |
| Age, years | 61.1 (12.6) | 60.4 (15.5) | 0.550 | 0.046 |
| Sex, male | 134 (70.2) | 378 (59.3) | 0.008 | 0.228 |
| Body mass index | 25.6 [22.9, 29.2] | 25.3 [22.8, 29.1] | 0.330 | 0.112 |
| Race, white | 171 (90.0) | 560 (88.5) | 0.602 | 0.049 |
| **ADMISSION DATA** |  |  |  |  |
| Hospital A | 121 (63.4) | 293 (46.0) | <0.001 | 0.354 |
| Admission type, surgical | 58 (30.4) | 177 (27.8) | 0.522 | 0.057 |
| SDD use during admission | 149 (78.0) | 438 (68.8) | 0.014 | 0.210 |
| **CHRONIC COMORBIDITIES** |  |  |  |  |
| Charlson Comorbidity Index (no age) | 2 [0, 4] | 2 [0, 3] | 0.141 | 0.106 |
| Any malignancy | 52 (27.2) | 137 (21.5) | 0.116 | 0.133 |
| Non-metastatic solid tumor | 27 (14.1) | 66 (10.4) | 0.152 | 0.115 |
| Metastatic malignancy | 7 (3.7) | 27 (4.2) | 0.837 | 0.029 |
| Hematologic malignancy | 21 (11.0) | 46 (7.2) | 0.098 | 0.131 |
| Diabetes mellitus (type 1 or type 2) | 36 (18.8) | 138 (21.7) | 0.420 | 0.070 |
| Cerebrovascular disease | 23 (12.0) | 58 (9.1) | 0.266 | 0.096 |
| Hemiplegia | 5 (2.6) | 24 (3.8) | 0.653 | 0.065 |
| **CHRONIC MEDICATION** |  |  |  |  |
| Any immunosuppressant | 28 (14.7) | 97 (15.8) | 0.819 | 0.030 |
| Antiplatelet drugs | 50 (26.3) | 160 (26.1) | >0.999 | 0.004 |
| Calcium-entry blockers | 36 (18.8) | 98 (15.4) | 0.264 | 0.091 |
| Beta-adrenergic blockers | 55 (28.8) | 167 (26.3) | 0.515 | 0.057 |
| Oral antidiabetic drugs | 22 (11.5) | 89 (14.0) | 0.468 | 0.074 |
| Insulin | 20 (10.5) | 68 (10.7) | >0.999 | 0.007 |
| **DISEASE SEVERITY AT ICU ADMISSION** |  |  |  |  |
| APACHE IV score | 90.6 (29.6) | 83.8 (28.8) | 0.005 | 0.233 |
| Acute physiology score | 77.7 (27.2) | 71.1 (26.8) | 0.003 | 0.246 |
| mSOFA score | 8 [6, 11] | 7 [5, 9] | <0.001 | 0.404 |
| Shock | 136 (72.3) | 331 (52.0) | <0.001 | 0.428 |
| ARDS | 56 (29.3) | 160 (25.1) | 0.260 | 0.095 |
| AKI | 95 (49.7) | 256 (40.2) | 0.024 | 0.193 |
| Gastrointestinal Failure score |  |  | 0.001 | 0.358 |
| 0 - Normal gastrointestinal function | 78 (41.3) | 363 (57.1) |  |  |
| 1 - Reduced/delayed enteral feeding† | 85 (45.0) | 186 (29.2) |  |  |
| 2 - Food intolerance or IAH | 25 (13.2) | 82 (12.9) |  |  |
| 3 - Food intolerance and IAH | 1 (0.5) | 3 (0.5) |  |  |
| 4 - Abdominal compartment syndrome | 0 (0.0) | 1 (0.2) |  |  |
| Gastrointestinal Failure score >= 1 | 111 (58.7) | 271 (42.7) | <0.001 | 0.324 |
| Gastrointestinal bleeding | 4 (2.1) | 11 (1.7) | 0.758 | 0.027 |
| Mechanically ventilated | 183 (97.3) | 546 (85.8) | <0.001 | 0.423 |
| PaO2/FiO2 ratio | 144 [93, 210] | 164 [110, 225] | 0.017 | 0.190 |
| **SOURCE OF INFECTION** |  |  |  |  |
| Pulmonary tract | 103 (53.9) | 323 (50.7) | 0.458 | 0.065 |
| Abdominal tract | 34 (17.8) | 118 (18.5) | 0.915 | 0.019 |
| Urinary tract | 16 (8.4) | 40 (6.3) | 0.325 | 0.081 |
| Cardiovascular | 9 (4.7) | 28 (4.4) | 0.843 | 0.015 |
| Skin | 12 (6.3) | 25 (3.9) | 0.166 | 0.107 |
| Central nervous system | 5 (2.6) | 35 (5.5) | 0.124 | 0.146 |
| Other or unknown | 25 (13.1) | 104 (16.3) | 0.307 | 0.092 |
| AKI = acute kidney injury; APACHE-IV = acute physiology and chronic health evaluation IV; ARDS = acute respiratory distress syndrome; IAH = intraabdominal hypertension; ICU = intensive care unit; mSOFA = modified sequential organ failure assessment score (without the central nervous system component); SMD = standardized mean difference.  *SMD >0.2 indicates a substantial imbalance between groups; <0.1 indicates a negligible difference  † “Enteral feeding <50% of calculated needs or no feeding 3 days after abdominal surgery” in the original paper.[18]  Categorical data are displayed as count (percentage) and compared using Fisher's exact test.  Normally distributed continuous data are displayed as mean (standard deviation) and compared using Welch's *t*-test.  Non-normally distributed continuous data are displayed as median [interquartile range] and compared using Wilcoxon's rank-sum test. | | | | |
|  |  |  |  |  |

***Supplementary Table 13. Baseline characteristics for the sensitivity analysis with an exposure period of 96 hours***

|  | **Erythromycin**  **(n = 242)** | **Controls**  **(n = 371)** | ***P-*value** | **SMD*** |
| --- | --- | --- | --- | --- |
| **DEMOGRAPHICS** |  |  |  |  |
| Age, years | 60.7 (13.6) | 60.9 (15.1) | 0.864 | 0.014 |
| Sex, male | 163 (67.4) | 218 (58.8) | 0.033 | 0.179 |
| Body mass index | 25.4 [22.9, 29.1] | 25.7 [22.9, 29.3] | >0.999 | 0.018 |
| Race, white | 215 (89.2) | 335 (90.3) | 0.682 | 0.036 |
| **ADMISSION DATA** |  |  |  |  |
| Hospital A | 154 (63.6) | 192 (51.8) | 0.005 | 0.242 |
| Admission type, surgical | 76 (31.4) | 81 (21.8) | 0.010 | 0.218 |
| SDD use during admission | 180 (74.4) | 252 (67.9) | 0.103 | 0.143 |
| **CHRONIC COMORBIDITIES** |  |  |  |  |
| Charlson Comorbidity Index (no age) | 2 [0, 4] | 2 [0, 3] | 0.157 | 0.088 |
| Any malignancy | 60 (24.8) | 85 (22.9) | 0.627 | 0.044 |
| Non-metastatic solid tumor | 35 (14.5) | 42 (11.3) | 0.264 | 0.094 |
| Metastatic malignancy | 6 (2.5) | 14 (3.8) | 0.488 | 0.074 |
| Hematologic malignancy | 23 (9.5) | 31 (8.4) | 0.663 | 0.040 |
| Diabetes mellitus (type 1 or type 2) | 50 (20.7) | 81 (21.8) | 0.763 | 0.029 |
| Cerebrovascular disease | 30 (12.4) | 39 (10.5) | 0.514 | 0.059 |
| Hemiplegia | 6 (2.5) | 14 (3.8) | 0.488 | 0.074 |
| **CHRONIC MEDICATION** |  |  |  |  |
| Any immunosuppressant | 36 (15.1) | 58 (16.2) | 0.733 | 0.030 |
| Antiplatelet drugs | 62 (25.9) | 94 (26.2) | 1.000 | 0.006 |
| Calcium-entry blockers | 43 (17.8) | 62 (16.8) | 0.744 | 0.027 |
| Beta-adrenergic blockers | 69 (28.5) | 96 (25.9) | 0.515 | 0.058 |
| Oral antidiabetic drugs | 30 (12.4) | 52 (14.1) | 0.628 | 0.049 |
| Insulin | 26 (10.7) | 41 (11.1) | >0.999 | 0.011 |
| **DISEASE SEVERITY AT ICU ADMISSION** |  |  |  |  |
| APACHE IV score | 91.5 (27.8) | 85.4 (28.1) | 0.008 | 0.219 |
| Acute physiology score | 78.7 (25.5) | 72.5 (26.1) | 0.004 | 0.239 |
| mSOFA score | 8 [6, 11] | 7 [5, 9] | <0.001 | 0.467 |
| Shock | 178 (74.2) | 199 (53.8) | <0.001 | 0.434 |
| ARDS | 86 (35.5) | 114 (30.7) | 0.219 | 0.102 |
| AKI | 123 (50.8) | 154 (41.5) | 0.025 | 0.188 |
| Gastrointestinal Failure score |  |  | <0.001 | 0.482 |
| 0 - Normal gastrointestinal function | 85 (35.1) | 215 (58.0) |  |  |
| 1 - Reduced/delayed enteral feeding† | 113 (46.7) | 104 (28.0) |  |  |
| 2 - Food intolerance or IAH | 42 (17.4) | 51 (13.7) |  |  |
| 3 - Food intolerance and IAH | 2 (0.8) | 1 (0.3) |  |  |
| 4 - Abdominal compartment syndrome | 0 (0.0) | 0 (0.0) |  |  |
| Gastrointestinal Failure score >= 1 | 157 (64.9) | 156 (42.0) | <0.001 | 0.470 |
| Gastrointestinal bleeding | 9 (3.7) | 4 (1.1) | 0.041 | 0.173 |
| Mechanically ventilated | 232 (96.7) | 337 (91.1) | 0.008 | 0.235 |
| PaO2/FiO2 ratio | 145 [99, 233] | 153 [102, 214] | 0.836 | 0.006 |
| **SOURCE OF INFECTION** |  |  |  |  |
| Pulmonary tract | 122 (50.4) | 208 (56.1) | 0.185 | 0.113 |
| Abdominal tract | 49 (20.2) | 64 (17.3) | 0.394 | 0.077 |
| Urinary tract | 18 (7.4) | 23 (6.2) | 0.620 | 0.049 |
| Cardiovascular | 13 (5.4) | 15 (4.0) | 0.437 | 0.063 |
| Skin | 14 (5.8) | 12 (3.2) | 0.152 | 0.123 |
| Central nervous system | 7 (2.9) | 19 (5.1) | 0.221 | 0.114 |
| Other or unknown | 39 (16.1) | 54 (14.6) | 0.645 | 0.043 |
| AKI = acute kidney injury; APACHE-IV = acute physiology and chronic health evaluation IV; ARDS = acute respiratory distress syndrome; IAH = intraabdominal hypertension; ICU = intensive care unit; mSOFA = modified sequential organ failure assessment score (without the central nervous system component); SMD = standardized mean difference.  *SMD >0.2 indicates a substantial imbalance between groups; <0.1 indicates a negligible difference  † “Enteral feeding <50% of calculated needs or no feeding 3 days after abdominal surgery” in the original paper.[18]  Categorical data are displayed as count (percentage) and compared using Fisher's exact test.  Normally distributed continuous data are displayed as mean (standard deviation) and compared using Welch's *t*-test.  Non-normally distributed continuous data are displayed as median [interquartile range] and compared using Wilcoxon's rank-sum test. | | | | |
|  |  |  |  |  |

***Supplementary Table 14. PS matched and PS weighted populations for the sensitivity analysis with an exposure period of 48 hours***

|  | **PS MATCHED** | | | **PS WEIGHTED** | | |
| --- | --- | --- | --- | --- | --- | --- |
|  | **Erythromycin**  **(n = 175)** | **Controls**  **(n = 175)** | **SMD*** | **Erythromycin** | **Controls** | **SMD*** |
| **DEMOGRAPHICS** |  |  |  |  |  |  |
| Age, years | 61.3 (12.7) | 62.7 (14.5) | 0.106 | 61.8 (12.9) | 60.8 (15.3) | 0.069 |
| Sex, male | 120 (68.6) | 115 (65.7) | 0.061 | 66.1 | 61.7 | 0.092 |
| Body mass index | 25.5 [23.0, 29.2] | 26.0 [23.4, 29.4] | 0.027 | 24.9 [22.8, 28.0] | 25.5 [22.8, 29.2] | 0.013 |
| Race, white | 155 (89.1) | 166 (95.4) | 0.238 | 87.2 | 89.7 | 0.080 |
| **ADMISSION DATA** |  |  |  |  |  |  |
| Hospital A | 109 (62.3) | 118 (67.4) | 0.108 | 54.3 | 51.1 | 0.063 |
| Admission type, surgical | 53 (30.3) | 51 (29.1) | 0.025 | 31.2 | 28.3 | 0.065 |
| SDD use during admission | 135 (77.1) | 131 (74.9) | 0.054 | 76.6 | 69.5 | 0.161 |
| **CHRONIC COMORBIDITIES** |  |  |  |  |  |  |
| Charlson Comorbidity Index (no age) | 2 [0, 3] | 2 [0, 3] | 0.030 | 2 [0, 3] | 2 [0, 3] | 0.003 |
| Any malignancy | 47 (26.9) | 44 (25.1) | 0.039 | 22.7 | 23.1 | 0.010 |
| Non-metastatic solid tumor | 23 (13.1) | 26 (14.9) | 0.049 | 11.8 | 11.2 | 0.017 |
| Metastatic malignancy | 7 (4.0) | 5 (2.9) | 0.063 | 3.5 | 4.4 | 0.048 |
| Hematologic malignancy | 20 (11.4) | 13 (7.4) | 0.137 | 8.4 | 7.7 | 0.024 |
| Diabetes mellitus (type 1 or type 2) | 32 (18.3) | 44 (25.1) | 0.167 | 18.6 | 22.4 | 0.095 |
| Cerebrovascular disease | 20 (11.4) | 20 (11.4) | <0.001 | 11.6 | 9.1 | 0.081 |
| Hemiplegia | 4 (2.3) | 7 (4.0) | 0.098 | 3.9 | 3.3 | 0.034 |
|  | |  |  |  |  |  |
| Any immunosuppressant | 26 (14.9) | 33 (19.4) | 0.119 | 13.8 | 16.4 | 0.071 |
| Antiplatelet drugs | 45 (25.9) | 48 (28.2) | 0.053 | 24.3 | 26.4 | 0.048 |
| Calcium-entry blockers | 34 (19.4) | 38 (21.7) | 0.057 | 20.4 | 15.3 | 0.133 |
| Beta-adrenergic blockers | 51 (29.1) | 47 (26.9) | 0.051 | 32.5 | 26.2 | 0.138 |
| Oral antidiabetic drugs | 20 (11.4) | 23 (13.1) | 0.052 | 11.4 | 13.9 | 0.074 |
| Insulin | 18 (10.3) | 26 (14.9) | 0.138 | 10.7 | 11.2 | 0.016 |
| **DISEASE SEVERITY AT ICU ADMISSION** | |  |  |  |  |  |
| APACHE IV score | 90.6 (30.2) | 92.9 (28.3) | 0.078 | 88.3 (30.1) | 85.7 (29.6) | 0.087 |
| Acute physiology score | 77.7 (27.8) | 79.0 (26.8) | 0.049 | 75.6 (27.6) | 72.8 (27.5) | 0.101 |
| mSOFA score | 8 [6, 11] | 8 [6, 10] | 0.044 | 7 [5, 10] | 7 [5, 9] | 0.057 |
| Shock | 126 (72.0) | 127 (72.6) | 0.013 | 59.1 | 56.5 | 0.052 |
| ARDS | 53 (30.3) | 51 (29.1) | 0.025 | 26.4 | 25.7 | 0.015 |
| AKI | 86 (49.1) | 89 (50.9) | 0.034 | 45.4 | 42.5 | 0.059 |
| Gastrointestinal Failure score |  |  | 0.104 |  |  | 0.103 |
| 0 - Normal gastrointestinal function | 76 (43.4) | 76 (43.4) |  | 48.5 | 53.2 |  |
| 1 - Reduced/delayed enteral feeding† | 75 (42.9) | 69 (39.4) |  | 36.5 | 31.8 |  |
| 2 - Food intolerance or IAH | 23 (13.1) | 29 (16.6) |  | 14.3 | 14.4 |  |
| 3 - Food intolerance and IAH | 1 (0.6) | 1 (0.6) |  | 0.7 | 0.6 |  |
| 4 - Abdominal compartment syndrome | 0 (0.0) | 0 (0.0) |  | 0.0 | 0.0 |  |
| Gastrointestinal Failure score >= 1 | 99 (56.6) | 99 (56.6) | <0.001 | 51.5 | 46.8 | 0.093 |
| Gastrointestinal bleeding | 3 (1.7) | 1 (0.6) | 0.108 | 2.2 | 1.6 | 0.043 |
| Mechanically ventilated | 170 (97.1) | 169 (96.6) | 0.033 | 93.8 | 89.0 | 0.175 |
| PaO2/FiO2 ratio | 144 [90, 212] | 153 [102, 223] | 0.105 | 161 [93, 236] | 158 [108, 222] | 0.066 |
| **SOURCE OF INFECTION** |  |  |  |  |  |  |
| Pulmonary tract | 96 (54.9) | 100 (57.1) | 0.046 | 51.2 | 52.0 | 0.018 |
| Abdominal tract | 31 (17.7) | 33 (18.9) | 0.030 | 20.7 | 18.3 | 0.061 |
| Urinary tract | 14 (8.0) | 16 (9.1) | 0.041 | 6.3 | 6.8 | 0.022 |
| Cardiovascular | 9 (5.1) | 7 (4.0) | 0.055 | 3.6 | 4.0 | 0.020 |
| Skin | 12 (6.9) | 6 (3.4) | 0.156 | 4.6 | 4.3 | 0.014 |
| Central nervous system | 3 (1.7) | 4 (2.3) | 0.041 | 3.3 | 4.7 | 0.073 |
| Other or unknown | 22 (12.6) | 19 (10.9) | 0.053 | 16.0 | 15.4 | 0.015 |
| AKI = acute kidney injury; APACHE-IV = acute physiology and chronic health evaluation IV; ARDS = acute respiratory distress syndrome; IAH = intraabdominal hypertension; ICU = intensive care unit; mSOFA = modified sequential organ failure assessment score (without the central nervous system component); SMD = standardized mean difference.  *SMD >0.2 indicates a substantial imbalance between groups; <0.1 indicates a negligible difference  † “Enteral feeding <50% of calculated needs or no feeding 3 days after abdominal surgery” in the original paper.[18] | | | | | | |

***Supplementary Table 15. PS matched and PS weighted populations for the sensitivity analysis with an exposure period of 96 hours***

|  | **PS MATCHED** | | | **PS WEIGHTED** | | |
| --- | --- | --- | --- | --- | --- | --- |
|  | **Erythromycin**  **(n = 211)** | **Controls**  **(n = 211)** | **SMD*** | **Erythromycin** | **Controls** | **SMD*** |
| **DEMOGRAPHICS** |  |  |  |  |  |  |
| Age, years | 60.7 (13.9) | 61.6 (14.3) | 0.069 | 61.5 (13.5) | 60.9 (14.7) | 0.042 |
| Sex, male | 139 (65.9) | 134 (63.5) | 0.050 | 65.2 | 62.5 | 0.057 |
| Body mass index | 25.4 [22.9, 29.1] | 25.7 [22.8, 29.2] | 0.012 | 25.5 [22.9, 28.8] | 25.7 [22.8, 29.3] | 0.002 |
| Race, white | 184 (87.6) | 197 (93.4) | 0.197 | 89.6 | 91.6 | 0.068 |
| **ADMISSION DATA** |  |  |  |  |  |  |
| Hospital A | 125 (59.2) | 124 (58.8) | 0.010 | 56.9 | 56.4 | 0.009 |
| Admission type, surgical | 60 (28.4) | 59 (28.0) | 0.011 | 25.6 | 25.6 | <0.001 |
| SDD use during admission | 154 (73.0) | 146 (69.2) | 0.084 | 73.8 | 68.4 | 0.121 |
| **CHRONIC COMORBIDITIES** |  |  |  |  |  |  |
| Charlson Comorbidity Index (no age) | 2.0 [0.0, 3.5] | 2.0 [0.0, 3.0] | 0.009 | 2.0 [0.0, 3.0] | 2.0 [0.0, 3.0] | 0.018 |
| Any malignancy | 52 (24.6) | 51 (24.2) | 0.011 | 22.9 | 23.8 | 0.022 |
| Non-metastatic solid tumor | 30 (14.2) | 22 (10.4) | 0.116 | 14.3 | 11.3 | 0.089 |
| Metastatic malignancy | 5 (2.4) | 13 (6.2) | 0.188 | 1.9 | 4.2 | 0.129 |
| Hematologic malignancy | 20 (9.5) | 18 (8.5) | 0.033 | 7.8 | 8.8 | 0.038 |
| Diabetes mellitus (type 1 or type 2) | 44 (20.9) | 46 (21.8) | 0.023 | 21.1 | 22.3 | 0.029 |
| Cerebrovascular disease | 27 (12.8) | 22 (10.4) | 0.074 | 11.8 | 10.1 | 0.056 |
| Hemiplegia | 5 (2.4) | 4 (1.9) | 0.033 | 3.1 | 3.1 | 0.002 |
|  | |  |  |  |  |  |
| Any immunosuppressant | 32 (15.4) | 36 (17.6) | 0.059 | 18.1 | 16.7 | 0.037 |
| Antiplatelet drugs | 56 (26.9) | 50 (24.4) | 0.058 | 23.8 | 26.4 | 0.060 |
| Calcium-entry blockers | 36 (17.1) | 34 (16.1) | 0.025 | 17.1 | 16.6 | 0.012 |
| Beta-adrenergic blockers | 61 (28.9) | 62 (29.4) | 0.010 | 28.5 | 26.1 | 0.054 |
| Oral antidiabetic drugs | 27 (12.8) | 28 (13.3) | 0.014 | 13.6 | 13.5 | 0.001 |
| Insulin | 23 (10.9) | 25 (11.8) | 0.030 | 11.9 | 12.2 | 0.008 |
| **DISEASE SEVERITY AT ICU ADMISSION** | |  |  |  |  |  |
| APACHE IV score | 90.5 (27.6) | 90.8 (29.4) | 0.014 | 89.9 (27.4) | 88.5 (29.6) | 0.052 |
| Acute physiology score | 77.6 (25.4) | 77.5 (27.5) | 0.004 | 76.7 (25.2) | 75.4 (27.4) | 0.052 |
| mSOFA score | 8 [6, 11] | 8 [6, 10] | 0.055 | 8 [5, 10] | 8 [5, 10] | 0.034 |
| Shock | 150 (71.1) | 147 (69.7) | 0.031 | 63.9 | 62.6 | 0.027 |
| ARDS | 76 (36.0) | 74 (35.1) | 0.020 | 32.5 | 32.0 | 0.011 |
| AKI | 104 (49.3) | 107 (50.7) | 0.028 | 47.6 | 45.8 | 0.035 |
| Gastrointestinal Failure score |  |  | 0.097 |  |  | 0.110 |
| 0 - Normal gastrointestinal function | 82 (38.9) | 83 (39.3) |  | 46.9 | 48.1 |  |
| 1 - Reduced/delayed enteral feeding† | 90 (42.7) | 84 (39.8) |  | 37.7 | 34.1 |  |
| 2 - Food intolerance or IAH | 37 (17.5) | 43 (20.4) |  | 14.7 | 17.5 |  |
| 3 - Food intolerance and IAH | 2 (0.9) | 1 (0.5) |  | 0.7 | 0.3 |  |
| 4 - Abdominal compartment syndrome | 0 (0.0) | 0 (0.0) |  | 0.0 | 0.0 |  |
| Gastrointestinal Failure score >= 1 | 129 (61.1) | 128 (60.7) | 0.010 | 53.1 | 51.9 | 0.023 |
| Gastrointestinal bleeding | 7 (3.3) | 1 (0.5) | 0.210 | 3.3 | 1.0 | 0.165 |
| Mechanically ventilated | 203 (96.2) | 206 (97.6) | 0.082 | 94.5 | 93.4 | 0.045 |
| PaO2/FiO2 ratio | 144 [96, 236] | 144 [95, 213] | 0.055 | 152 [99, 237] | 148 [100, 210] | 0.106 |
| **SOURCE OF INFECTION** |  |  |  |  |  |  |
| Pulmonary tract | 109 (51.7) | 110 (52.1) | 0.009 | 51.9 | 53.5 | 0.032 |
| Abdominal tract | 44 (20.9) | 43 (20.4) | 0.012 | 19.8 | 18.9 | 0.025 |
| Urinary tract | 16 (7.6) | 16 (7.6) | <0.001 | 6.7 | 6.6 | 0.003 |
| Cardiovascular | 12 (5.7) | 11 (5.2) | 0.021 | 4.3 | 4.4 | 0.004 |
| Skin | 10 (4.7) | 8 (3.8) | 0.047 | 4.2 | 4.4 | 0.011 |
| Central nervous system | 6 (2.8) | 7 (3.3) | 0.027 | 3.4 | 4.0 | 0.032 |
| Other or unknown | 32 (15.2) | 30 (14.2) | 0.027 | 16.0 | 14.6 | 0.039 |
| AKI = acute kidney injury; APACHE-IV = acute physiology and chronic health evaluation IV; ARDS = acute respiratory distress syndrome; IAH = intraabdominal hypertension; ICU = intensive care unit; mSOFA = modified sequential organ failure assessment score (without the central nervous system component); SMD = standardized mean difference.  *SMD >0.2 indicates a substantial imbalance between groups; <0.1 indicates a negligible difference  † “Enteral feeding <50% of calculated needs or no feeding 3 days after abdominal surgery” in the original paper.[18] | | | | | | |

***Supplementary Table 16. Hazard ratios for mortality up to day 90 for the sensitivity analysis with an exposure period of 48 hours***

|  | **90-day mortality rate** | |
| --- | --- | --- |
| **UNADJUSTED** | **Events, n (%)** | **HR (95% CI)** |
| Erythromycin (n = 191) | 63 (34.2) | 0.93 (0.70 - 1.23) |
| Controls (n = 637) | 227 (36.7) | 1.00 (ref) |
| **PS MATCHED** |  |  |
| Erythromycin (n = 175) | 57 (33.9) | 0.79 (0.56 - 1.12) |
| Controls (n = 175) | 70 (41.4) | 1.00 (ref) |
| **PS WEIGHTED** | **Events, %** |  |
| Erythromycin | 35.8 | 0.94 (0.65 – 1.37) |
| Controls | 38.2 | 1.00 (ref) |
| CI = confidence interval; HR = hazard ratio; IPTW = inverse probability of treatment weighting; PS = propensity score; ref = referent. | | |

***Supplementary Table 17. Hazard ratios for mortality up to day 90 for the sensitivity analysis with a grace period of 96 hours***

|  | **90-day mortality rate** | |
| --- | --- | --- |
| **UNADJUSTED** | **Events, n (%)** | **HR (95% CI)** |
| Erythromycin (n = 242) | 83 (35.3) | 0.93 (0.71 - 1.23) |
| Controls (n = 371) | 138 (38.3) | 1.00 (ref) |
| **PS MATCHED** |  |  |
| Erythromycin (n = 211) | 70 (34.1) | 0.89 (0.64 - 1.24) |
| Controls (n = 211) | 78 (38.2) | 1.00 (ref) |
| **PS WEIGHTED** | **Events, %** |  |
| Erythromycin | 36.2 | 0.96 (0.70 - 1.31) |
| Controls | 38.9 | 1.00 (ref) |
| CI = confidence interval; HR = hazard ratio; IPTW = inverse probability of treatment weighting; PS = propensity score; ref = referent. | | |

***Supplementary Table 18. Secondary clinical outcomes for the sensitivity analysis with a grace period of 48 hours***

|  | **UNADJUSTED** | | | **PS MATCHED** | | | **PS WEIGHTED** | | |
| --- | --- | --- | --- | --- | --- | --- | --- | --- | --- |
|  | **Erythromycin  (n = 191)** | **Controls  (n = 637)** | ***P-*value** | **Erythromycin  (n = 175)** | **Controls  (n = 175)** | ***P-*value** | **Erythromycin** | **Controls** | ***P-*value** |
| **SECONDARY CLINICAL OUTCOMES** | | | |  | | |  | | |
| 30-day mortality | 51 (27.1) | 166 (26.5) | 0.851 | 47 (27.3) | 51 (29.8) | 0.727 | 27.7 | 27.9 | 0.955 |
| ICU length of stay, days | 7 [4, 12] | 5 [3, 9] | <0.001 | 7 [4, 11] | 6 [3, 10] | 0.173 | 6 [4, 11] | 5 [3, 9] | 0.007 |
| Hospital length of stay, days | 20 [11, 36] | 17 [9, 33] | 0.094 | 20 [12, 36] | 18 [10, 32] | 0.509 | 20 [10, 34] | 17 [9, 32] | 0.355 |
| Duration of mechanical ventilation, days | 5 [3, 9] | 4 [2, 7] | <0.001 | 5 [3, 9] | 4 [2, 8] | 0.034 | 5 [2, 9] | 4 [2, 8] | 0.044 |
| ΔSOFA day 4 | -1.7 (2.8) | -1.2 (2.6) | 0.106 | -1.7 (2.9) | -1.6 (2.6) | 0.759 | -1.5 (2.7) | -1.4 (2.7) | 0.636 |
| Incidence of ICU-acquired infections | 25 (13.1) | 51 (8.0) | 0.044 | 22 (12.6) | 19 (10.9) | 0.877 | 8.8 | 8.8 | 0.974 |
| Incidence of ICU-acquired AKI | 9 (4.7) | 24 (3.8) | 0.532 | 9 (5.1) | 4 (2.3) | 0.228 | 4.0 | 4.0 | 0.976 |
| Incidence of ICU-acquired ARDS | 5 (2.6) | 19 (3.0) | >0.999 | 5 (2.9) | 5 (2.9) | >0.999 | 1.8 | 3.3 | 0.228 |
| AKI = acute kidney injury; ARDS = acute respiratory distress syndrome; ICU = intensive care unit; PS = propensity score; ΔSOFA = change in sequential organ failure assessment score from admission to day 2, 3, or 4.  Categorical data are displayed as count (percentage) or and compared using Fisher's exact test (unadjusted) or McNemar's test (after PS matching), or displayed as percentage and compared using a chi-square test† (after PS weighting).  Normally distributed continuous data are displayed as mean (standard deviation) and compared using a *t*-test (unadjusted), a paired *t*-test (after PS matching) or a *t*-test† (after PS weighting).  Non-normally distributed continuous data are displayed as median [interquartile range] and compared using Wilcoxon's rank-sum test (unadjusted), Wilcoxon's signed-rank test, or Wilcoxon rank-sum test† (after PS weighting).  †for weighted samples, as provided in the *survey* R package | | | | | | | | | |

***Supplementary Table 19. Secondary clinical outcomes for the sensitivity analysis with a grace period of 96 hours***

|  | **UNADJUSTED** | | | **PS MATCHED** | | | **PS WEIGHTED** | | |
| --- | --- | --- | --- | --- | --- | --- | --- | --- | --- |
|  | **Erythromycin  (n = 242)** | **Controls  (n = 371)** | ***P-*value** | **Erythromycin  (n = 211)** | **Controls  (n = 211)** | ***P-*value** | **Erythromycin** | **Controls** | ***P-*value** |
| **SECONDARY CLINICAL OUTCOMES** | | | |  | | |  | | |
| 30-day mortality | 64 (26.8) | 93 (25.4) | 0.706 | 52 (25.0) | 53 (25.6) | 0.913 | 27.6 | 26.0 | 0.694 |
| ICU length of stay, days | 9 [6, 15] | 8 [5, 12] | 0.054 | 9 [6, 15] | 8 [6, 13] | 0.440 | 8 [6, 14] | 8 [6, 13] | 0.858 |
| Hospital length of stay, days | 24 [13, 43] | 21 [13, 39] | 0.191 | 24 [14, 43] | 21 [12, 39] | 0.286 | 21 [12, 39] | 22 [13, 39] | 0.943 |
| Duration of mechanical ventilation, days | 7 [4, 11] | 6 [4, 10] | 0.061 | 7 [4, 12] | 7 [4, 10] | 0.309 | 7 [4, 11] | 7 [4, 10] | 0.569 |
| ΔSOFA day 4 | -1.4 (2.9) | -1.2 (2.6) | 0.412 | -1.3 (3.0) | -1.4 (2.8) | 0.716 | -1.2 (2.8) | -1.4 (2.7) | 0.439 |
| Incidence of ICU-acquired infections | 46 (19.0) | 51 (13.7) | 0.090 | 38 (18.0) | 37 (17.5) | >0.999 | 15.1 | 15.6 | 0.874 |
| Incidence of ICU-acquired AKI | 18 (7.4) | 24 (6.5) | 0.629 | 16 (7.6) | 15 (7.1) | >0.999 | 6.5 | 6.6 | 0.930 |
| Incidence of ICU-acquired ARDS | 10 (4.1) | 19 (5.1) | 0.698 | 9 (4.3) | 16 (7.6) | 0.230 | 3.4 | 5.6 | 0.214 |
| AKI = acute kidney injury; ARDS = acute respiratory distress syndrome; ICU = intensive care unit; PS = propensity score; ΔSOFA = change in sequential organ failure assessment score from admission to day 2, 3, or 4.  Categorical data are displayed as count (percentage) or and compared using Fisher's exact test (unadjusted) or McNemar's test (after PS matching), or displayed as percentage and compared using a chi-square test† (after PS weighting).  Normally distributed continuous data are displayed as mean (standard deviation) and compared using a *t*-test (unadjusted), a paired *t*-test (after PS matching) or a *t*-test† (after PS weighting).  Non-normally distributed continuous data are displayed as median [interquartile range] and compared using Wilcoxon's rank-sum test (unadjusted), Wilcoxon's signed-rank test, or Wilcoxon rank-sum test† (after PS weighting).  †for weighted samples, as provided in the *survey* R package | | | | | | | | | |

***Supplementary Table 20. Cause-specific and subdistribution hazard ratios for ICU discharge and mortality for treatment with erythromycin***

|  | **90-day mortality rate** | | |
| --- | --- | --- | --- |
| **UNADJUSTED** | **Cause-specific HR (95% CI)** | | **Subdistribution HR (95% CI)** |
| ICU discharge | 0.76 (0.64 - 0.91) | | 0.79 (0.67 - 0.93) |
| Mortality | 0.97 (0.69 - 1.38) | | 1.25 (0.89 - 1.76) |
| **PS MATCHED** |  |  | |
| ICU discharge | 1.02 (0.83 - 1.26) | | 0.99 (0.80 - 1.21) |
| Mortality | 0.97 (0.65 - 1.46) | | 0.96 (0.64 - 1.43) |
| CI = confidence interval; HR = hazard ratio; PS = propensity score | | | |

**SUPPLEMENTARY FIGURES**

***Supplementary Figure 1. Flowchart of patients used in estimating the PS and subsequent***
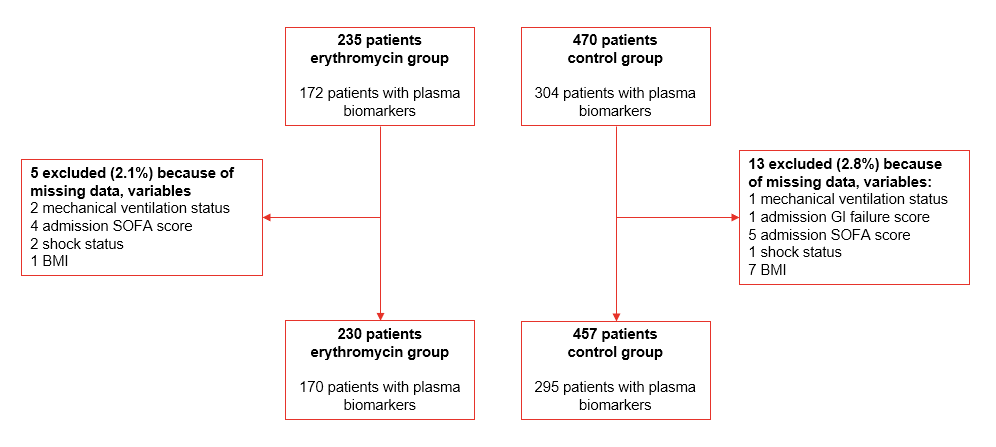
***analyses (complete cases)***

BMI = body mass index; GI = gastrointestinal; SOFA = sequential organ failure assessment score

##
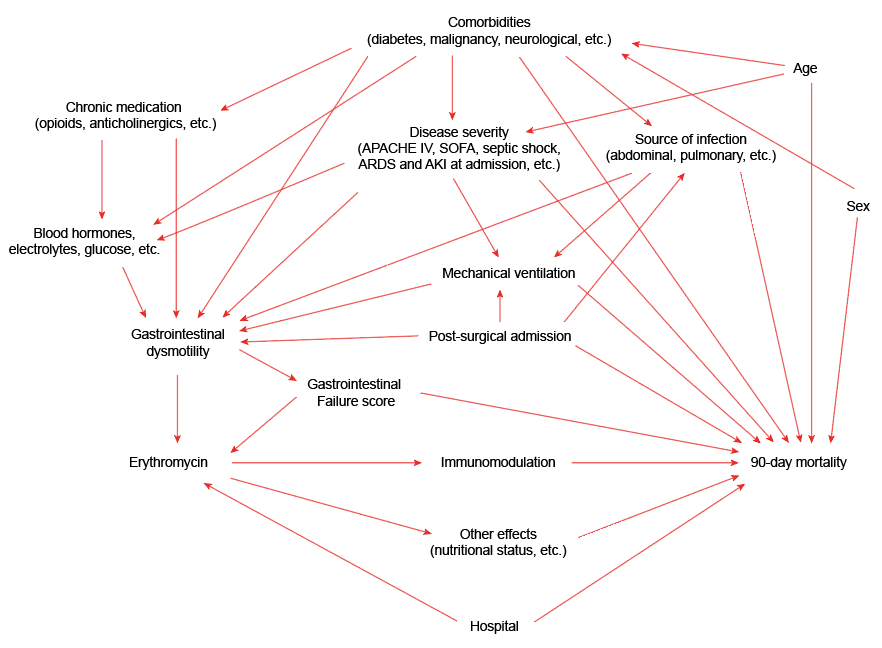
Supplementary Figure 2. Proposed directed acyclic graph

Relationship between the exposure (treatment with erythromycin to alleviate gastrointestinal dysmotility) and the primary outcome (90-day mortality), and the baseline covariates that affect this relationship. AKI = acute kidney injury; APACHE-IV = Acute Physiology And Chronic Health Evaluation IV score; ARDS = acute respiratory distress syndrome; SOFA = sequential organ failure assessment score.

***
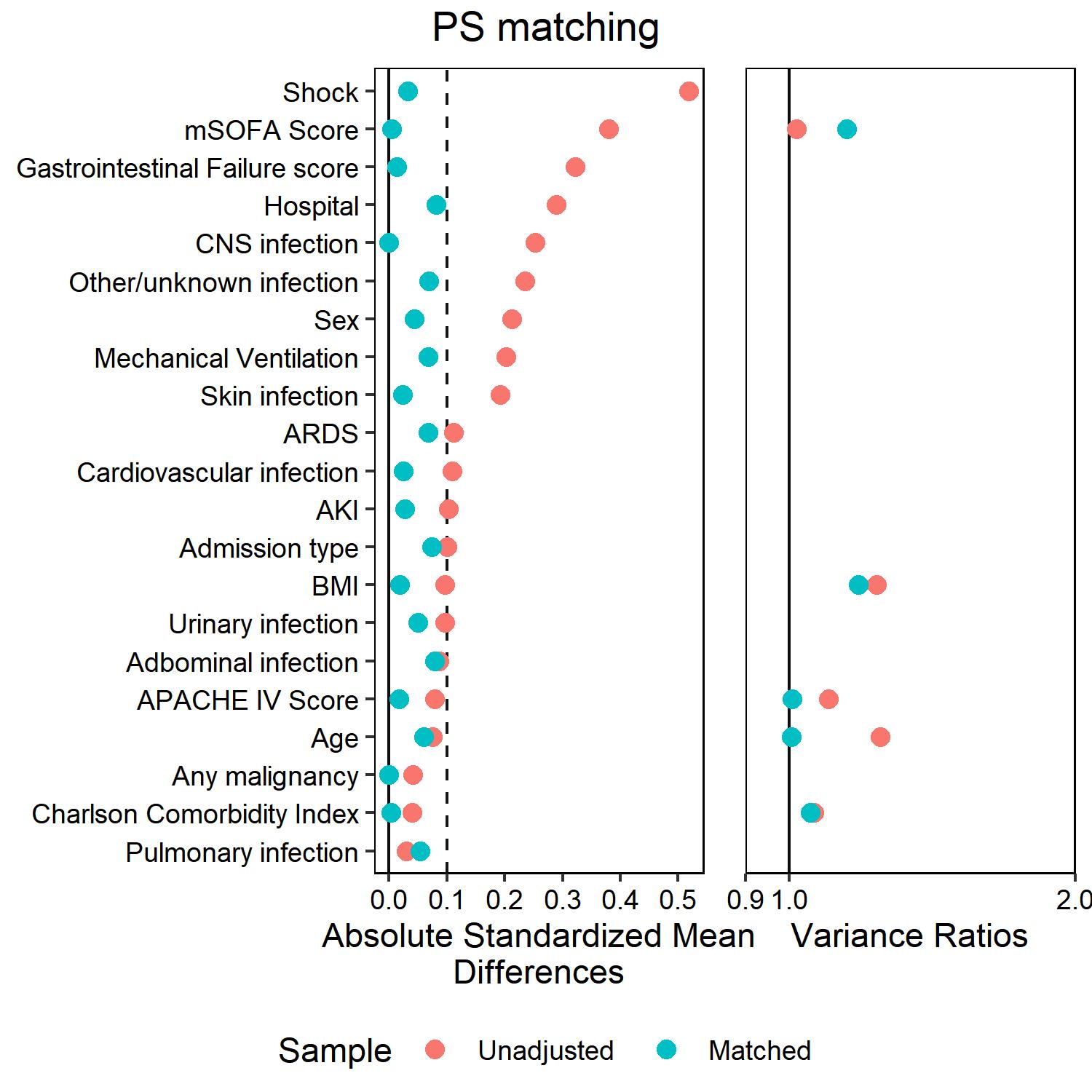
Supplementary Figure 3. Balance statistics of the covariates used for PS matching in the population with measured biomarkers***

Plot depicting the absolute SMDs and absolute variance ratios between the unadjusted and the propensity score matched population for the covariates used in the model to estimate the propensity scores.

## Supplementary Figure 4. IL-8/IL-10 and IL-6/IL-10 ratios in PS matched treated (n = 150) and control (n = 150) patients


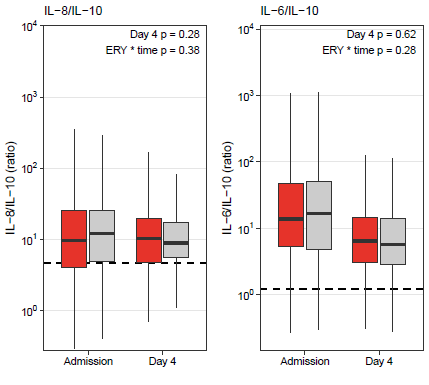


The box represents the 25th percentile, median and 75th percentile. The whiskers represent up to 1.5 times the interquartile range. The dashed line represents the median value in healthy volunteers. The p-values are derived from linear mixed models using log2-transformed biomarkers as the dependent variable and including a random slope and intercept for the change over time per patient. “Day 4 p” is the difference between groups at day 4 (obtained as the p-value for the treatment coefficient in models using day 4, rather than admission, as the reference category). “ERY * time p” is the interaction term for treatment and time i.e. whether the slope over time (from admission to day 4) differs between groups. ERY = erythromycin.

*
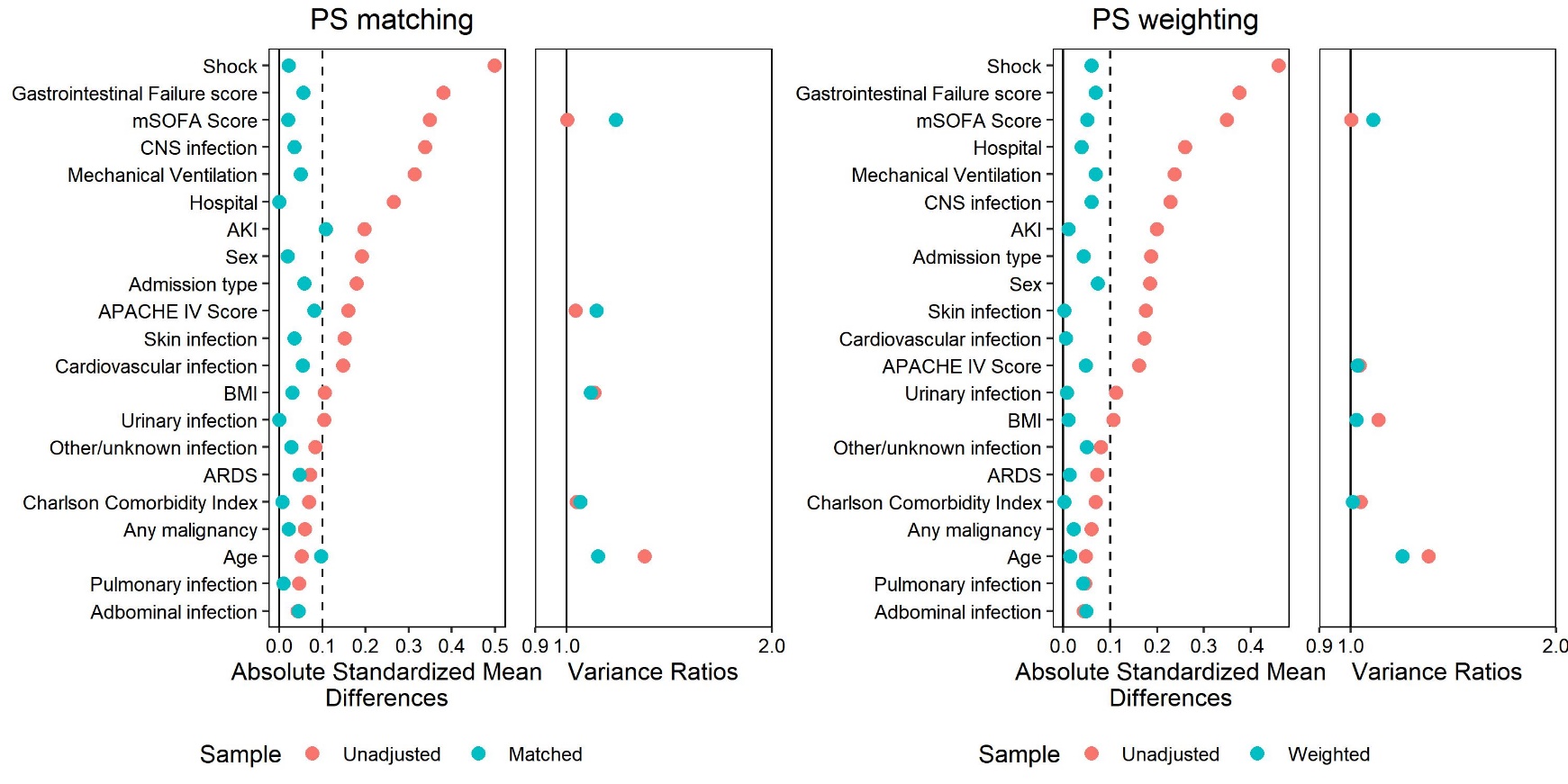
****Supplementary Figure 5. Balance statistics of the covariates used for PS matching and weighting for the intention-to-treat sensitivity analysis***

Plot depicting the absolute SMDs and absolute variance ratios between the unadjusted and the propensity score matched or propensity score weighted populations for the covariates used in the model to estimate the propensity scores.

***
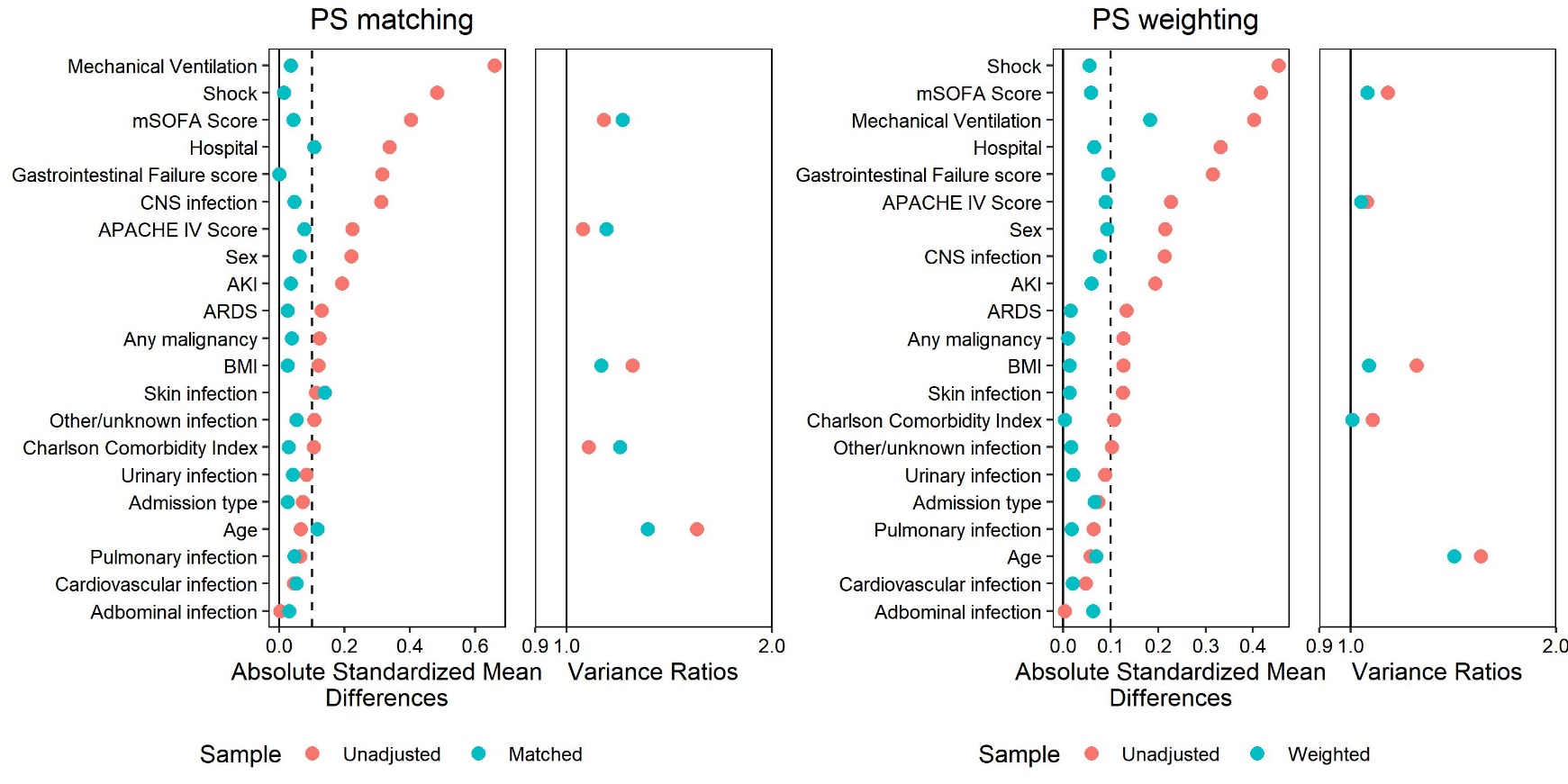
Supplementary Figure 6. Balance statistics of the covariates used for PS matching and weighting for the sensitivity analysis with an exposure period of 48 hours***

Plot depicting the absolute SMDs and absolute variance ratios between the unadjusted and the propensity score matched or propensity score weighted populations for the covariates used in the model to estimate the propensity scores.

***Supplementary Figure 7. Balance statistics of the covariates used for PS matching and weighting for the sensitivity analysis with an exposure period of 96 hours***

***
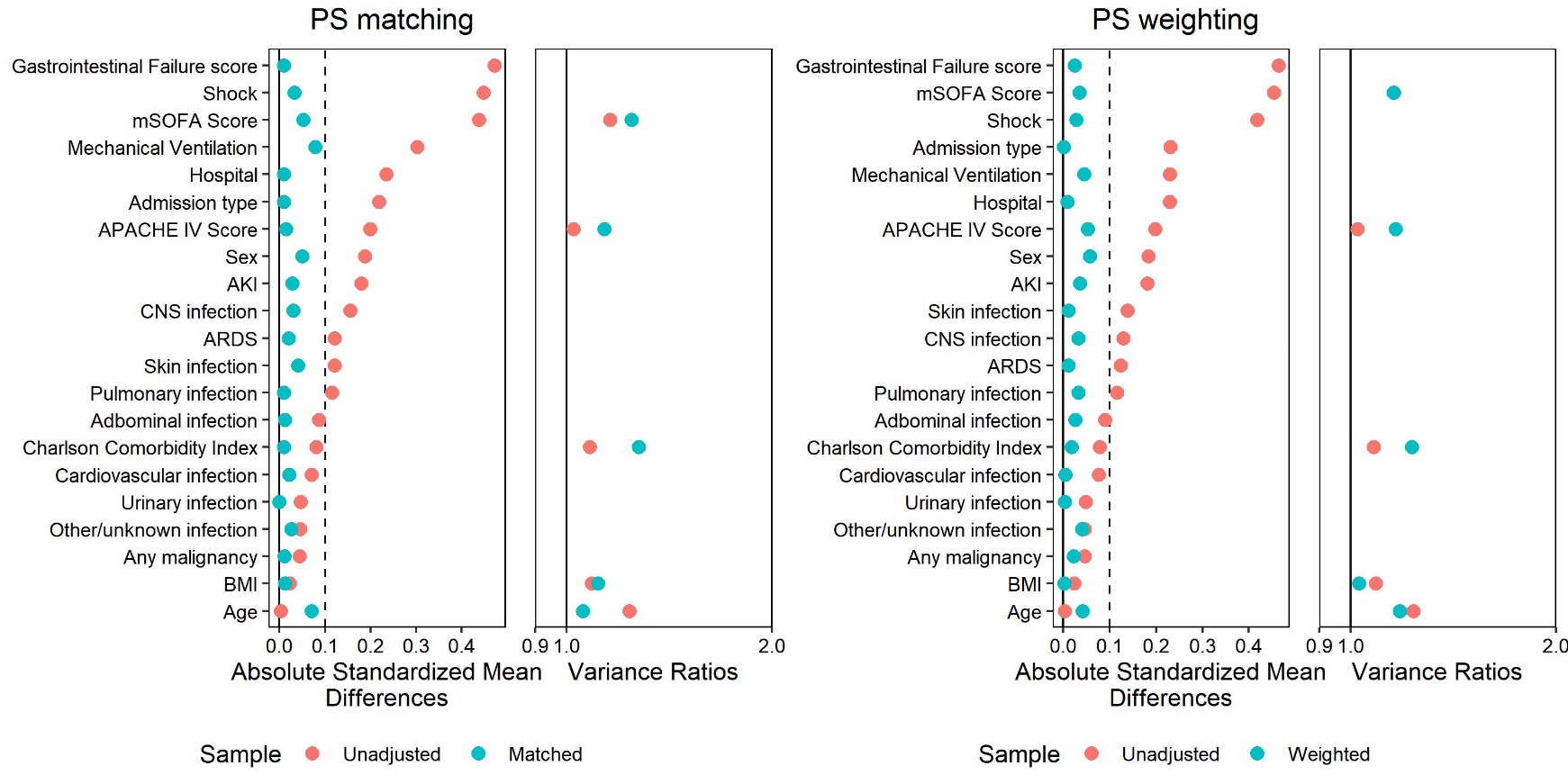
***

Plot depicting the absolute SMDs and absolute variance ratios between the unadjusted and the propensity score matched or propensity score weighted populations for the covariates used in the model to estimate the propensity scores.

# REFERENCES

1. Klouwenberg PMCK, Ong DSY, Bos LDJ, De Beer FM, Van Hooijdonk RTM, Huson MA, et al. Interobserver agreement of centers for disease control and prevention criteria for classifying infections in critically ill patients. Critical Care Medicine. 2013;41:2373–8.

2. Van Vught LAV, Klouwenberg PMCK, Spitoni C, Scicluna BP, Wiewel MA, Horn J, et al. Incidence, risk factors, and attributable mortality of secondary infections in the intensive care unit after admission for sepsis. JAMA - Journal of the American Medical Association. 2016;315:1469–79.

3. Scicluna BP, Klein Klouwenberg PMC, van Vught LA, Wiewel MA, Ong DSY, Zwinderman AH, et al. A molecular biomarker to diagnose community-acquired pneumonia on intensive care  unit admission. American journal of respiratory and critical care medicine. United States; 2015;192:826–35.

4. Uhel F, Peters-Sengers H, Falahi F, Scicluna BP, van Vught LA, Bonten MJ, et al. Mortality and host response aberrations associated with transient and persistent acute kidney injury in critically ill patients with sepsis: a prospective cohort study. Intensive Care Medicine. Springer Berlin Heidelberg; 2020;46:1576–89.

5. Bellomo R, Ronco C, Kellum JA, Mehta RL, Palevsky P. Acute renal failure - definition, outcome measures, animal models, fluid therapy and  information technology needs: the Second International Consensus Conference of the Acute Dialysis Quality Initiative (ADQI) Group. Critical care (London, England). 2004;8:R204-12.

6. Bernard GR, Artigas A, Brigham KL, Carlet J, Falke K, Hudson L, et al. Report of the American-European consensus conference on ARDS: Definitions, mechanisms, relevant outcomes and clinical trial coordination. Intensive Care Medicine. 1994;20:225–32.

7. Ranieri VM, Rubenfeld GD, Thompson BT, Ferguson ND, Caldwell E, Fan E, et al. Acute respiratory distress syndrome: The Berlin definition. JAMA - Journal of the American Medical Association. 2012;307:2526–33.

8. Simonis FD, de Iudicibus G, Cremer OL, Ong DSY, van der Poll T, Bos LD, et al. Macrolide therapy is associated with reduced mortality in acute respiratory distress  syndrome (ARDS) patients. Annals of translational medicine. 2018;6:24.

9. Charlson ME, Pompei P, Ales KL, MacKenzie CR. A new method of classifying prognostic comorbidity in longitudinal studies:  development and validation. Journal of chronic diseases. England; 1987;40:373–83.

10. Lumley T. Analysis of Complex Survey Samples. Journal of Statistical Software; Vol 1, Issue 8 (2004). 2004;

11. Van Vught LA, Wiewel MA, Hoogendijk AJ, Frencken JF, Scicluna BP, Klouwenberg PMCK, et al. The host response in patients with sepsis developing intensive care unit-acquired secondary infections. American Journal of Respiratory and Critical Care Medicine. 2017;196:458–70.

12. Van Vught LA, Scicluna BP, Wiewel MA, Hoogendijk AJ, Klouwenberg PMCK, Franitza M, et al. Comparative analysis of the host response to community-acquired and hospital-acquired pneumonia in critically ill patients. American Journal of Respiratory and Critical Care Medicine. 2016;194:1366–74.

13. Van Der Weele TJ, Ding P. Sensitivity analysis in observational research: Introducing the E-Value. Annals of Internal Medicine. 2017;167:268–74.

14. Mathur MB, Ding P, Riddell CA, VanderWeele TJ. Web Site and R Package for Computing E-values. Epidemiology (Cambridge, Mass). 2018;29:e45–7.

15. Fine JP, Gray RJ. A Proportional Hazards Model for the Subdistribution of a Competing Risk. Journal of the American Statistical Association [Internet]. Taylor & Francis; 1999;94:496–509. Available from: https://www.tandfonline.com/doi/abs/10.1080/01621459.1999.10474144

16. Austin PC. The use of propensity score methods with survival or time-to-event outcomes: Reporting measures of effect similar to those used in randomized experiments. Statistics in Medicine. 2014;33:1242–58.

17. Austin PC, Fine JP. Propensity-score matching with competing risks in survival analysis. Statistics in Medicine. John Wiley and Sons Ltd; 2019;38:751–77.

18. Reintam A, Parm P, Kitus R, Starkopf J, Kern H. Gastrointestinal Failure score in critically ill patients: A prospective observational study. Critical Care. 2008;12:1–8.
